# Serine supplementation suppresses hypoxia-induced pathological retinal angiogenesis

**DOI:** 10.7150/thno.105299

**Published:** 2025-04-09

**Authors:** Hitomi Yagi, Chenxi Qiu, Yan Zeng, Myriam Boeck, Shen Nian, Chuck T. Chen, Jarrod C Harman, Taku Kasai, Jeff Lee, Victoria Hirst, Katherine Neilsen, Chaomei Wang, Kiran Bora, Meenakshi Maurya, Tori Rodrick, Matthew Grumbine, Yuelin Yang, Zhanqing Hua, Ian R. Sweet, Sasha A. Singh, Masanori Aikawa, Jing Chen, Zhongjie Fu

**Affiliations:** 1 Department of Ophthalmology, Boston Children's Hospital, Harvard Medical School, Boston, MA 02115, USA.; 2 Department of Ophthalmology, Keio University School of Medicine, 160-8582 Tokyo, Japan.; 3 Department of Medicine, Beth Israel Deaconess Medical Center, Harvard Medical School, Boston, MA 02115, USA.; 4 Department of Neurology, Beth Israel Deaconess Medical Center, Harvard Medical School, Boston, MA 02115, USA.; 5 Eye Center, Medical Center, Faculty of Medicine, University of Freiburg, 79106 Freiburg, Germany.; 6 Department of Pathology, Xi'an Medical University, Shaanxi Province, 710021, China.; 7 Department of Nutritional Sciences, Temerty Faculty of Medicine, University of Toronto, Toronto, Ontario, Canada.; 8 Center for Interdisciplinary Cardiovascular Sciences, Division of Cardiovascular Medicine, Department of Medicine, Brigham Women's Hospital, Harvard Medical School, Boston, MA 02115, USA.; 9 Metabolomics Core Resource Laboratory, NYU Langone Health, New York, NY 10016, USA.; 10 EnTox Sciences, Inc., Mercer Island, WA 98040, USA.; 11 University of Washington Medicine Diabetes Institute, University of Washington, Seattle, WA 98109, USA.; 12 Center for Excellence in Vascular Biology, Division of Cardiovascular Medicine, Brigham and Women's Hospital, Harvard Medical School, Boston, MA 02115, USA.; 13 Channing Division of Network Medicine, Department of Medicine, Brigham Women's Hospital, Harvard Medical School, Boston, MA 02115, USA.

**Keywords:** serine, retina, angiogenesis, neovascularization, retinopathy of prematurity

## Abstract

**Rationale**: Pathological retinal angiogenesis with irregular and fragile vessels (also termed neovascularization, a response to hypoxia and dysmetabolism) is a leading cause of vision loss in all age groups. This process is driven in part through the energy deficiency in retinal neurons. Sustaining neural retinal metabolism with adequate nutrient supply may help prevent vision-threatening neovascularization. Low circulating serine levels are associated with neovascularization in macular telangiectasia and altered serine/glycine metabolism has been suggested in retinopathy of prematurity. We here explored the role of serine metabolism in suppressing hypoxia-driven retinal neovascularization using oxygen-induced retinopathy (OIR) mouse model.

**Methods**: We administered wild-type C57BL/6J OIR pups with systemic serine or provided the nursing dam with a serine/glycine-deficient diet during the relative hypoxic phase, followed by analysis of retinal vasculature at postnatal (P) 17, the time of peak neovascularization. Retinas from P17 OIR pups with either systemic serine supplementation or vehicle control were subjected to metabolomics, lipidomics, proteomics, and single-cell RNA sequencing. To validate the role of mitochondrial fatty acid oxidation (FAO) and oxidative phosphorylation (OXPHOS) in mediating serine protection against OIR, we treated OIR pups with inhibitors to block FAO or OXPHOS along with either serine or vehicle. The potential transcriptional mediator and pro-angiogenic signals were validated by western blotting.

**Results**: Systemic serine supplementation reduced retinal neovascularization, while maternal dietary serine/glycine deficiency exacerbated it. FAO was essential in mediating serine protective effects, and serine supplementation increased levels of phosphatidylcholine, the most abundant phospholipids in the retina. Serine treatment a) increased the abundance of proteins involved in OXPHOS in retinas, b) increased the expression of mitochondrial respiration-related genes, and c) decreased the expression of pro-angiogenic genes in rod photoreceptor cluster. Serine suppression of retinal neovascularization was dependent on mitochondrial energy production. High mobility group box 1 protein (HMGB1) was identified as a potential key mediator of serine suppression of pro-angiogenic signals in hypoxic retinas.

**Conclusions**: Our findings suggest that serine supplementation may serve as a potential therapeutic approach for neovascular eye diseases by enhancing retinal mitochondrial metabolism and lipid utilization, suppressing the key drivers of uncontrolled angiogenesis.

## Introduction

Pathological retinal neovessel growth is a leading cause of blindness. Neovascularization occurs as a response to hypoxia and dysmetabolism in conditions such as retinopathy of prematurity (ROP) in preterm infants and diabetic retinopathy (DR) in adults. In these diseases, the neural retina—particularly the photoreceptors—becomes hypoxic and deprived of nutrients. This metabolic stress triggers the growth of new retinal blood vessels to meet the metabolic energy demands of retina 1, 2. However, these newly formed vessels are irregular and fragile. Current treatments, including laser photocoagulation and anti-vascular endothelial growth factor (VEGF) therapy, have limitations and long-term safety concerns 3, 4. Therefore, a deeper understanding of the underlying disease mechanisms is necessary to develop new and safe therapeutic approaches. Photoreceptors (rods and cones) are rich in mitochondria which are important to provide the energy to maintain visual function. Oxidative phosphorylation (OXPHOS) in mitochondria is essential in maintaining neural retinal function. Disruption in mitochondrial function can lead to abnormal retinal neuronal responses, macular dystrophy, and subretinal deposits 5, 6. We have recently observed decreased mitochondrial respiration in mouse retinas with hypoxia-induced neovascularization and supplementing pyruvate (a key mitochondrial energy substrate) attenuated neovessel growth 7. We here aimed to explore potential nutrients that could improve retinal mitochondrial function and suppress neovascularization.

Premature infants, particularly those born at lower gestation ages and with a higher risk of ROP, exhibit lower circulating levels of amino acids (e.g., serine, glutamate, and alanine) 8. Metabolomics studies have identified alterations in serine, glycine, and related metabolic pathways, with elevated blood glycine levels in premature infants with ROP versus without ROP 9, 10. A systemic review of nine metabolic studies on patients with DR revealed significant changes in amino acid and energy metabolism compared to controls 11. Altered metabolism of glycine and serine, along with arginine and proline metabolism, and pyruvate metabolism, were among the top pathways identified in proliferative DR via vitreous metabolomics profiling 12. Increased retinal levels of amino acids such as serine, glycine, alanine, and threonine have also been observed in mouse retinas with neovascularization modeling hypoxia-induced proliferative ROP and DR 12, further suggesting a correlation between these amino acids and neovascularization. However, the precise role of these increased levels remains unclear. Low levels of circulating serine caused by genetic mutations of phosphoglycerate dehydrogenase (*PHGDH,* the first enzyme in endogenous serine synthesis by converting glycolytic metabolites) are associated with macular degeneration and subretinal neovascularization in humans 13, 14. In mice, dietary restriction of serine and glycine caused functional defects in the mouse retina, whereas dietary supplementation of high serine rescued retinal pathology 15. These observations suggest that serine deficiency impairs neurovascular retinal function, and that the availability of dietary serine modulates retinal health.

Our current study tested whether serine supplementation decreased the development of hypoxia-induced retinal neovascularization in mouse oxygen-induced retinopathy (OIR). In mouse OIR, neonatal mice are initially exposed to hyperoxia (75% oxygen), leading to retinal vessel loss. After the mouse neonates are returned to room air, relative hypoxia (from 75% oxygen to room air 21% oxygen) induces retinal neovessel growth (**Figure S1A**) 16. We examined the impact of 1) systemic serine supplementation and 2) maternal dietary deficiency of serine/glycine on the development of retinal neovascularization in hypoxic retinas. Given that serine depletion and turnover occur rapidly in circulation 17, it was technically challenging to measure changes in retinal serine levels in mouse pups with the intervention dose (0.6 µg/g from P12 to P16). Continuous ^13^C-labeled serine infusion (2, 10 nmol/min/g for 2 h) in fasted mice showed a high enrichment of serine in the retina, suggesting that the retina predominantly obtained serine from the circulation 15. Recent reports have also shown that 2-to-3-month dietary restriction of serine and glycine in mice alters plasma serine levels but not retinal serine levels 15, 18. Corresponding to these findings, we did not observe significant changes in retinal serine levels in OIR mice with the nursing dam fed a serine and glycine-deficient diet (data not shown). Despite these limitations, we found that serine supplementation suppressed pathological retinal neovessel growth through modulating retinal lipid use, thereby improving retinal health and decreasing the driving force of pro-angiogenic signals in photoreceptors.

## Methods

### Study Approval

All animal studies adhered to the Association for Research in Vision and Ophthalmology Statement for the Use of Animals in Ophthalmic and Vision Research and were approved by the Institutional Animal Care and Use Committee at Boston Children's Hospital (#00001619).

### Mouse model of oxygen-induced retinopathy (OIR)

Neonatal C57BL/6J mice (wild-type, Jackson Laboratory, #000664) with their nursing dam were exposed to 75% oxygen from postnatal day (P) 7 for five days 16 (**Figure S1A**). At P12, the mice were returned to room air. Hyperoxia causes retinal vessel loss between P7 to P12, and relative hypoxia (after P12) causes both retinal re-vascularization (reflected by decreased vaso-obliteration) and neovascularization. Neovascularization starts to form around P14 and reaches the maximum at P17 19. Retinas were collected for examination of the retinal vascular network, proteins, and RNA. Retinal vaso-obliteration and neovascularization were quantified in isolectin GS-IB4 (vessel marker, I21413, Invitrogen)-stained retinal whole mounts using Image J 16. The vaso-obliterated area in the central retina was identified by the absence of red fluorescent signals, while the neovascular area was characterized by an accumulation of high-intensity red fluorescent signals. Both the vaso-obliterated and neovascular areas were quantified and expressed as percentages of the total retinal area in the whole mount. The fold change was calculated by comparing the percentage values of treatments to the average value of the littermate vehicle controls. At least 2-3 independent litters were used for each treatment. Both male and female mice were used.

### Metabolite treatments

For intraperitoneal (i.p.) serine treatment, the littermate OIR mouse pups were randomly assigned to either L-serine (0.6 µg/g body weight, S4311, Sigma, dissolved into phosphate-buffered saline, PBS) or vehicle control (PBS) groups. The dose was chosen based on estimation from circulating serine levels in mice (100 µM, **Figure S1B**). A lower and higher dose of serine at 0.06 µg/g or 6 µg/g was also tested. OIR mice were treated daily from P7 to P11 (hyperoxia) or P12 to P16 (relative hypoxia). For oral serine intake, the littermate OIR mice were given either serine (0.6 or 6 µg/g body weight) or vehicle control (PBS) by oral gavage from P12 to P16. At P12 and P17, retinas were collected, and body weight was recorded. Alanine (2 μg/g body weight, i.p., A7627, Sigma), which differs from serine in that a hydroxyl group is replaced by methylenic hydrogens and can replace serine increasing cytotoxic lipid synthesis in serine deficient conditions 20, as well as glycine (0.8 µg/g body weight, i.p., #G5417, Sigma), which can be converted to serine endogenously 21, were also tested. For drug cotreatment, all pups were i.p. injected with etomoxir (4 µg/g body weight 22, #828934-41-4, Sigma,) or malonyl-CoA (15 µg/g body weight, #16455, Cayman) blocking fatty acid oxidation (FAO) with half treated with serine (0.6 µg/g body weight, i.p.) and half with PBS from P12 to P16. A similar strategy was also used for mitochondrial ATP synthase inhibitor oligomycin (0.25 µg/g body weight, i.p., P12 to P16 23, #11342, Cayman. A higher dose at 0.5 µg/g body weight caused increased mortality) or the inhibitor of high mobility group box 1 protein (HMGB1), glycyrrhizin (25 ug/g, i.p., #11847, Cayman. A higher dose at 50 µg/g body weight caused increased mortality) cotreatment with serine or PBS.

### Exogenous serine deficiency

To examine the impact of serine deficiency on OIR, we fed the nursing dam with serine/glycine-deficient (TD. 150056, Envigo Teklad Diets) 15 and control diets (TD. 110839, Envigo Teklad Diets) (a gift from Dr. Marin Gantner, Lowy Medical Research Institute) from P14 when retinal neovessel growth begins. Glycine was removed to prevent endogenous conversion of glycine to serine 21 to ensure serine deficiency. The diet composition is provided in **Supplementary Table 1 and 2.** At P17, retinas were collected for the analysis of vascular status.

### DL-Serine assay

Serums from OIR and non-OIR mice were collected at P17. Serum levels of total serine and D-serine were measured using a fluorometric DL-serine assay kit (#K743-100; BioVision) according to the manufacturer's protocols. Assay was conducted using a 96-well microplate immediately after sample collection. The values of L-serine were detected by subtracting D-serine levels from total DL-serine levels. Values were expressed as μM.

### Metabolomics

*Sample collection:* P17 OIR retinas were collected from L-serine (0.6 µg/g, i.p.)- vs. vehicle control-treated mice from P12 to P16. Six retinas were pooled as n = 1. n = 3 per group was used for metabolomics analysis by the NYU Metabolomics Core Resource Laboratory, New York, NY, USA. Raw data of metabolomics was deposited at Metabolomics Workbench 24 (Metabolomics of L-serine vs. vehicle control retinas: study_id: ST003512, datatrack_id: 5186, Project DOI: http://dx.doi.org/10.21228/M8T24J).

*Extraction of metabolites from retinas:* Prior to extraction, samples were moved from -80 °C storage to wet ice and thawed. Extraction buffer, consisting of 80% methanol (Fisher Scientific) and 500 nM metabolomics amino acid mix standard (Cambridge Isotope Laboratories, Inc.), was prepared and placed on dry ice. Samples were extracted at a concentration of 10 mg retina per 1 mL of prepared extraction buffer in 2.0 mL screw cap vials containing ~100 µL of disruption beads (Research Products International, Mount Prospect, IL). Each was homogenized for 10 cycles on a bead blaster homogenizer (Benchmark Scientific, Edison, NJ). Cycling consisted of a 30 s homogenization time at 6 m/s followed by a 30 s pause. Samples were subsequently spun at 21,000 g for 3 min at 4 °C. A set volume of each (450 µL) was transferred to a 1.5 mL tube and dried down by speedvac (Thermo Fisher, Waltham, MA). Samples were reconstituted in 50 µL of Optima liquid chromatography-mass spectrometry (LC-MS) grade water (Fisher Scientific, Waltham, MA). Samples were sonicated for 2 min, then spun at 21,000 g for 3 min at 4 °C. Twenty microliters were transferred to LC vials containing glass inserts for analysis. The remaining sample was placed in -80 °C for long term storage.

*LC-MS/MS with the polar untargeted metabolomics method:* Samples were subjected to an LCMS analysis to detect and quantify known peaks. A metabolite extraction was carried out on each sample based on a previously described method 25. The LC column was a Waters^TM^ Atlantis Premier Beh Z-HILIC Column, 1.7 µm, 2.1 mm X 150 mm coupled to a Dionex Ultimate 3000^TM^ system and the column oven temperature was set to 25 °C for the gradient elution. A flow rate of 100 μL/min was used with the following solvent A) 10 mM ammonium carbonate in water, pH 9.0, and solvent B) neat acetonitrile. The gradient profile was as follows; 80-20%B (0-30 min), 20-80%B (30-31 min), 80-80%B (31-42 min). Injection volume was set to 2 μL for all analyses (42 min total run time per injection).

MS analyses were carried out by coupling the LC system to a Thermo Q Exactive HF^TM^ mass spectrometer operating in heated electrospray ionization mode (HESI). Method duration was 30 min with a polarity switching data-dependent Top 5 method for both positive and negative ion modes. Spray voltage for both positive and negative modes was 3.5 kV and capillary temperature was set to 320 °C with a sheath gas rate of 35, aux gas of 10, and max spray current of 100 μA. The full MS scan for both polarities utilized 120,000 resolution with an AGC target of 3e6 and a maximum IT of 100 ms, and the scan range was from *m*/*z* 67-1000. Tandem MS spectra for both positive and negative mode used a resolution of 15,000, AGC target of 1e5, maximum IT of 50 ms, isolation window of 0.4 m/z, isolation offset of 0.1 m/z, fixed first mass of 50 m/z, and 3-way multiplexed normalized collision energies (nCE) of 10, 35, 80. The minimum AGC target was 1e4 with an intensity threshold of 2e5. All data were acquired in profile mode.

### Lipidomics

P17 OIR retinas were collected from L-serine (0.6 µg/g, i.p.)- vs. vehicle control-treated mice from P12 to P16. Eight retinas were pooled as n = 1. n = 3 per group was used for lipidomics analysis conducted at Mass Spectrometry Core Facility, University of Wisconsin-Madison. Total lipids from each sample of pooled retina were isolated by MTBE extraction 26 with internal standard mix containing Avanti SPLASH LipidoMix (#330707-1EA) at 10 µL per sample, C18 ceramide-d7 (d18:1-d7/18:0, #22788, Cayman Chemical), EOS-d9 (d18:1-d9/32:0/18:2, #24423, Cayman Chemical), linoleic acid-d11 (Cayman chemical, #9002193), and heptadecanoyl-L-carnitine-d3 (#35459, Cayman Chemical). Total lipids were analyzed by UHPLC-MS and UHPLC-MS/MS in both positive and negative ion modes. The UHPLC was an Agilent model 1290 Infinity II with G7120A binary pump, model G7167B multisampler, model G7116B column compartment, and model G7110B isocratic pump. The HPLC was connected to the inlet port of an Agilent G6546A QTOF mass spectrometer with an Agilent JetStream dual ESI source. UHPLC conditions remain the same in all data acquisitions. The column was a Waters Acquity UPLC BEH C18 1.7 µm 2.1 mm x 50 mm, with a guard column containing the same stationary phase with dimensions 2.1 mm x 5 mm. The solvents were solvent A) 10 mM ammonium formate, 0.1% (v/v) formic acid, 60% (v/v) acetonitrile in water and solvent B) 10 mM ammonium formate, 0.1% (v/v) formic acid, 9% (v/v) acetonitrile, 1% (v/v) water in 2-propanol. Column flow was kept at a constant 0.5 ml/min. Separation were performed with the following binary gradient: starting at 85% A and 15% B for 2.4 min, then 70% A and 30% B for 0.6 min, 52% A and 48% B for 10.2 min, 18% A and 82% B for 0.6 min, 1% A and 99% B for 2.2 min, and finally 85% A and 15% B for 4.6 min for a total run time of 20 min. Column temperature was set at 50 °C and samples were kept at 8 °C on the autosampler until injection. Injection volumes were set at 2 μL for positive ion mode MS, 5 μL for negative ion mode MS, 4 μL for positive ion mode MS/MS, and 7 μL for negative ion mode MS/MS. Lipid Annotator (Agilent software) was used to identify lipids based on mass and retention time. Dominant compositions consist of enumerated specific lipid acyl chain and degree of unsaturation, whereas sum compositions indicate lipid class, total carbons and unsaturation. Following assignment of lipid identity, Profinder (Agilent software) was used to extract and integrate ion chromatograms for each lipid species in each sample LC-MS data file. The percentage of each lipid class over total detected lipids in each sample was calculated and compared between serine- vs. vehicle-treated OIR retinas.

### BaroFuse analysis

*Assessment of oxygen-consumption rate (OCR,* reflective of mitochondria respiration*) in ex vivo retina:* OCR was measured in P17 C57BL/6J OIR retinas using a multichannel fluidics system that continuously bathes tissue with freshly oxygenated buffer containing precise levels of nutrients and test compounds, while simultaneously measuring real-time OCR (BaroFuse, BF-OXY-FC-8, EnTox Sciences Inc, Seattle, WA) 27. The BaroFuse instrument consists of two reservoirs of media each perfusing 4 tissue chambers allowing comparisons between two different protocols of test compound exposure run in parallel. Following instrument setup and equilibration between the gas in the head space of the reservoir (5% CO_2_/21% O_2_/balance N_2_) and the buffer (Krebs-Ringer Bicarbonate Solution with 20 mM HEPES (#J67795.K2, Sigma) and 1.1 mM glucose supplemented with 0.1% BSA (#A9647, Millipore)), experiments were performed by loading and perfusing a single retina per channel. Typically, retinas were loaded into three of the four chambers on each side, while the channel without tissue was used as control to correct the tissue data for time-dependent changes in the inflow O_2_. To decrease the biological variability, the two retinas from each mouse were placed in tissue chamber that were perfused with media from opposing reservoirs. After loading the tissue, retinas were equilibrated in the system for 90 min at a flow rate of ~ 20-25 mL/min to establish baseline OCRs. Following baseline measurements, responses in OCR to a glucose transport inhibitor 7 (20 µM BAY-876, #HY-100017, Medchemexpress LLC) measured for 120 min, were assessed in the following groups: Krebs-Ringer Solution with 1.1 mM glucose (control), Krebs-Ringer Solution (1.1 mM Glucose) supplemented with 10 mM serine, and Krebs-Ringer Solution (1.1 mM glucose) supplemented with 10 mM serine + 5 mM malate + 1 mM L-leucine + 1 mM glutamine as a positive control to evaluate the feasibility of the experimental design in identifying mitochondrial fuel substrates. The mixture was adapted from mito-fuel mixture (10 mM pyruvate, 5 mM malate, 1 mM glutamine, and 1mM leucine), which maintains OCR in mouse retinas *ex vivo* under the condition of glucose deprivation as previously published 28. After 120 min, a 3 mM injection of potassium cyanide (KCN, #60178, Sigma) was used to inhibit respiration in order to determine the inflow O2 for each channel's sensor. To calculate OCR for each channel (in nmol/min/retina), the following equation was used:

OCR = ([O_2_]_in_ - ([O_2_]_out_) × flow rate/retina

where flow rate is in mL/min and [O_2_] is in nmol/mL, and ([O_2_]_in_. was the [O_2_] determined in the presence of KCN for each channel. Fractional change of OCR was calculated relative to the baseline OCR. Calculations were made and graphs were generated with software that comes with the BaroFuse (BaroFuse Data Processor, EnTox Sciences).

### Proteomics

OIR mice were treated with L-serine (0.6 µg/g, i.p.) and vehicle (PBS) from P12 to P16. P17 OIR retinas from both eyes of one mouse (n = 8 mice for vehicle control and n = 8 for serine) were pooled and homogenized in RIPA buffer (#89900, Invitrogen) supplemented with 1% protease inhibitor and 0.1% phosphatase inhibitor. Lysates were proteolyzed using the iST in-solution digestion kit (PreOmics GmbH, Germany) automated on the PreON robot (PreOmics): 50 µg retina protein sample per 10 µL was added to 40 µL LYSE buffer (PreOmics). The samples were trypsinized for 3 h following the manufacturer's instructions. Eluted peptides were dried in a speed vacuum (EppendorfVacufuge) and resuspended in 40 µL LC-load.

Mass spectra were acquired on Orbitrap Fusion Lumos coupled to an Easy-nLC1000 HPLC pump (Thermo Fisher Scientific). The peptides were diluted 5-fold using LC-load and 4 µL injections separated using a dual column set-up: an Acclaim™ PepMap™ 100 C18 HPLC Column, 75 µm x 70 mm (#164946, Thermo Fisher Scientific); and an EASY-Spray™ HPLC Column, 75 µm x 250 mm (#ES902, Thermo Fisher Scientific). The column was heated at a constant temperature of 45 ˚C. The gradient flow rate was 300 nL/min from 5 to 21% solvent B (0.1% formic acid in acetonitrile) for 75 min, 21 to 30% solvent B for 15 min, and another 10 min of a 95%-5% solvent B in a jigsaw wash. Solvent A was 0.1% formic acid in mass spectrometry-grade water. The mass spectrometer was operated in positive mode. MS resolution was set to 120,000, and the top N precursor ions in a 3-second cycle time (within a scan range of m/z 400-1500; isolation window, 1.6 m/z) were subjected to collision-induced dissociation (CID, collision energy 30%) for peptide sequencing.

The acquired peptide spectra, comprising 16 retinal samples (n = 8 mice for vehicle control and n = 8 mice for serine), were searched with the Proteome Discoverer package (PD, version 2.5) using the SEQUEST-HT search algorithm against the Mouse UniProt database (63,603 entries, updated January 2022). The digestion enzyme was set to trypsin and up to two missed cleavages were allowed. The precursor tolerance was set to 10 ppm and the fragment tolerance window to 0.6 Da. Methionine oxidation and n-terminal acetylation were set as dynamic modifications, and cysteine carbamidomethylation as a static modification. The PD Percolator algorithm calculated the peptide false discovery rate (FDR) and peptides were filtered based on an FDR threshold of 1.0%. Peptides that were only assigned to one given protein group and not detected in any other protein group were considered unique and used for further analyses. A minimum of 2 unique peptides for each protein were required for the protein to be included in the analyses. The Feature Mapper was enabled in PD to quantify peptide precursors detected in the MS1 but may not have been sequenced in all samples. Chromatographic alignment was performed with a maximum retention time shift of 10 min, mass tolerance of 10 ppm and signal-to-noise minimum of 5. Precursor peptide abundances were based on their chromatographic intensities and total peptide amount was used for PD normalization.

Data were further analyzed using the statistical software, Qlucore (Qlucore, Sweden, version 3.5). We performed a two-group comparison (serine vs. vehicle control) using the log-transformed protein group means, the student's t-test for each protein's comparison (P-value), and the Benjamini-Hochberg procedure to calculate the FDR adjusted P-value. Proteins with significantly statistically increased or decreased abundance with P < 0.05 (adjusted p < 0.41) were inputted into the Enrichr database 29, 30 for gene ontology (GO) term analysis of biological processes. Enrichr provided P values computed from the Fisher's exact test, and adjusted P value was computed using the Benjamini-Hochberg method for correction for multiple hypotheses testing. Pathways were considered as significant with adjusted P < 0.05. The mass spectrometry proteomics data have been deposited to the ProteomeXchange Consortium via the PRIDE 31 partner repository with the dataset identifier PXD056490 and 10.6019/PXD056490.

### Single-cell transcriptomics (10x)

P17 OIR retinas were collected from L-serine (0.6 µg/g, i.p.)- vs. vehicle control-treated mice from P12 to P16. P17 OIR retinas from both eyes of one mouse (n = 4 mice for vehicle control and n = 4 mice for serine) were pooled. Single-cell suspensions were prepared from mouse retinas by using the Worthington papain dissociation system. 10X transcriptome library was prepared at the Single Cell Core at Harvard Medical School (HMS), Boston, MA, where 5000 cells per sample were barcoded, and 3' gene expression libraries were made from them following standard Chromium Next GEM Single Cell 3' Reagent Kits v3.1 (Dual Index) User Guide from 10X Genomics. Briefly, the single cells were encapsulated and barcoded within nanoliter-sized droplets along with the reagents necessary for cell lysis and reverse transcription. Within the droplet, cells are lysed, and the mRNA molecules are captured and barcoded during reverse transcription. Subsequently, full-length cDNA was made, followed by enzymatic fragmentation. P5, P7, i7, and i5 sample indexes and TruSeq Read 2 (read two primer sequences) were added via End Repair, A-tailing, Adaptor Ligation, and PCR. The libraries were sequenced for PE150 (151+10+10+151 cycles) on NovaSeq 6000 S4. Data were demultiplexed and converted to counts table using cellrange with built-in default parameters. Raw reads are deposited to SRA (PRJNA941894) in the fastq format.

The cellranger output was imputed and processed by Seurat 32, 33. Given the feature distribution, cells with less than 200 features (low-quality cells) or more than 6000 features (potential multiplets) were filtered out. Cells with more than 5% of mitochondrial transcripts were also filtered out. Data were log normalized using the Seurat built-in NormalizeData function with a scale factor 10000. Two thousand variable features were identified using the vst method of the Seurat built-in FindVariableFeatures function. All the features were scaled using the ScaleData function, and the identified variable features were used for principal component analysis. Thirty principal components were used for finding clusters and UMAP analyses, with an empirically determined resolution of 0.4. Potential doublets were further computationally removed by DoubletFinder 34.

Cell types were identified and provisionally assigned by comparing the expression profile of each individual cells to the reference profile from the mouse cell atlas 35. Clusters with mixed populations of cell types, defined by having more than 20% of at least two cell types, were filtered for clarity. The identity of each cluster is independently validated by examining the expression of known markers. Clusters of the same major cell types were subsequently combined for further analyses, following common practice. Differential gene expression analyses were computed using the MAST test using Seurat. Enrichment analyses of the pathways were performed using Enrichr 30. Average expression for each animal and cell type were computed and scaled using Seurat, and the heatmaps were visualized and presented using Morpheus (https://software.broadinstitute.org/morpheus/).

### Western blot

P17 OIR retinas isolated from serine (0.6 µg/g, i.p.)- vs. vehicle control (PBS)-treated OIR mice were homogenized in RIPA buffer (#89900, Invitrogen) supplemented with 1% protease inhibitor and 0.1% phosphatase inhibitor. After centrifugation of retinal lysates, the protein concentration was quantified with the BCA assay (23225, Thermo Scientific). 40 ug proteins were subjected to SDS-PAGE gel electrophoresis and transferred to 0.2 μm pore-size PVDF membranes. Primary antibodies against HMGB1 (1:1000, #PA1-16926, Invitrogen), HIF-1α (1:1000, #NB100-479, Novus Biologicals), phospho-STAT3 (Tyr705) (1:1000, #9131, Cell Signaling), STAT3 (79D7) (1:1000, #4904, Cell Signaling), and β-ACTIN (1:1000, #A1978, Sigma) were used. HRP-conjugated anti-rabbit antibody and HRP-conjugated anti-mouse antibody were used as secondary antibodies (1:10000). West pico plus sensitivity chemiluminescent (ECL) substrate (#34579, Thermo Scientific) for HMGB1, HIF-1α, STAT3 (79D7), β-ACTIN, or West femto maximum ECL substrate (#34094, Thermo Scientific) for phospho-STAT3 (Tyr705) were used to visualize the signals. Images were taken with the Azure 600 (Azure Biosystems), and band densities were quantified using ImageJ.

### Real-time quantitative PCR (RT-qPCR)

P17 OIR retinas were isolated from serine (0.6 µg/g, i.p.)- vs. vehicle control (PBS)-treated OIR mice. Total RNA was extracted from pooled retinas of both eyes with PureLinkTM RNA Mini Kit (#12183025, Invitrogen), and cDNA was generated with iScriptTM Reverse Transcription Supermix (#1708841, Bio-Rad). RT-qPCR was performed using SYBR Green qPCR Master Mix (#522076, Bimake.com) and CFX96TM Real-Time PCR Detection System (Bio-Rad, California, USA). Data were quantified using the ∆∆Ct method with Cyclophilin A (*CypA*) (F: 5' - CAG ACG CCA CTG G - 3'; R: 5' - TGT CTT TGG AAC TTT GTC TGC AA - 3') as the internal control. The sequences of primer were *Hmgb1* (F: 5' - GCA TCC TGG CTT ATC CAT TGG - 3'; R: 5' - GGC TGC TTG TCA TCT GCT G - 3'). The relative value of *Hmgb1* was divided by that of *CypA* for each sample. Fold change was calculated referring to the average relative value of control groups.

### Statistics

Statistics were included under the figure legends. For non-omics datasets, normality (histogram, quantile-quantile (QQ) plot, and Shapiro-Wilk test) and variance (F-test) were confirmed. Parametric unpaired t-test for the data with normal distribution and equal variance (or Welch's t-test for the data with unequal variance) or non-parametric Mann-Whitney test for the data without normal distribution was used to compare the groups (Prism v10.0; GraphPad Software, Inc., San Diego, CA). *P* < 0.05 was considered as statistically significant.

## Results

### Serine supplementation decreased and dietary serine/glycine deficiency worsened hypoxia-induced retinal neovascularization

**Figure 1:**
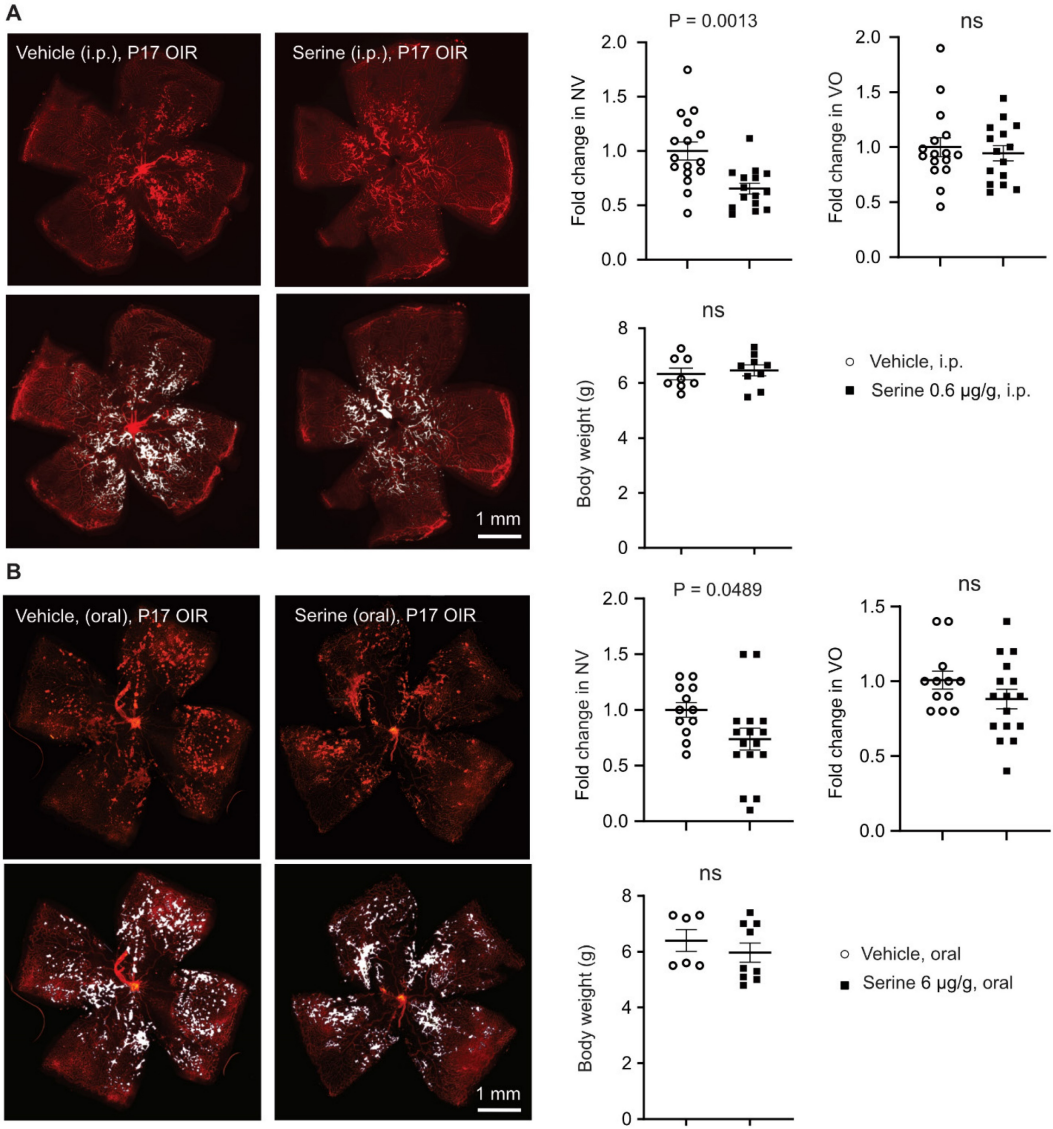
** Serine supplementation during the hypoxic phase of OIR decreased retinal neovascularization.** Serine or vehicle was delivered by i.p. injection (0.6 µg/g, **A**) or oral gavage (6 µg/g) (**B**) to mouse littermates from P12 to P16 (relative hypoxia). At P17, retinal vaso-obliteration (VO, central area without red fluorescence) and neovascularization (NV, mid-peripheral area with high intensity of red fluorescence, highlighted in white) were examined. Body weight was monitored. n = 16-17 retinas per group (**A**), n = 12-16 retinas per group (**B**). Scale bar, 1 mm. Fold change was calculated relative to the average value of littermate vehicle controls. Unpaired t-test (or Welch's t-test) or Mann-Whitney test was used to compare the groups. ns, not significant.

Our previous report showed that serine levels in the retina were higher in OIR than in non-OIR (normal controls) mice 12. We also observed accumulated serine concentration in the plasma in OIR vs. non-OIR mice at P17 (**Figure S1B**). To assess the role of accumulated serine in OIR on retinal vascularization, we used serine supplementation and depletion approaches. We first supplemented serine to mouse OIR pups via two systemic approaches (0.6 µg/g, i.p., or 6 µg/g, oral gavage). Both routes of serine treatment during the relative hypoxic phase (P12 to P16) decreased retinal neovascularization and had no impact on retinal re-vascularization (reflected by vaso-obliterated area) (**Figure 1A-B**). Serine supplementation (0.6 µg/g, i.p.) during hyperoxia (P7 to P11) did not reduce hyperoxia-induced retinal vessel loss at P12 (**Figure S1C**). A lower dose of serine (0.06 µg/g, i.p.) also suppressed neovascularization, but the effect was milder than that of serine (0.6 µg/g, i.p.) (**Figure S1D**). A higher dose of serine (6 µg/g, i.p.) did not show a significant impact on OIR pathology (**Figure S1D**). Oral administration of serine at a lower dose (0.6 µg/g, oral) did not protect against OIR (**Figure S1E**). These observations suggested that serine supplementation targeted pathological retinal angiogenesis and did not affect physiological retinal vessel regrowth under relative hypoxia in OIR. Additionally, we tested other amino acids closely associated with serine, such as alanine and glycine. Alanine treatment during hypoxia did not significantly impact retinal vasculature in OIR mice (**Figure S2A**). Glycine supplementation during hyperoxia and hypoxia decreased retinal vaso-obliteration (**Figure S2B-C**), suggesting glycine-promoted physiological retinal vessel regrowth.

**Figure 2:**
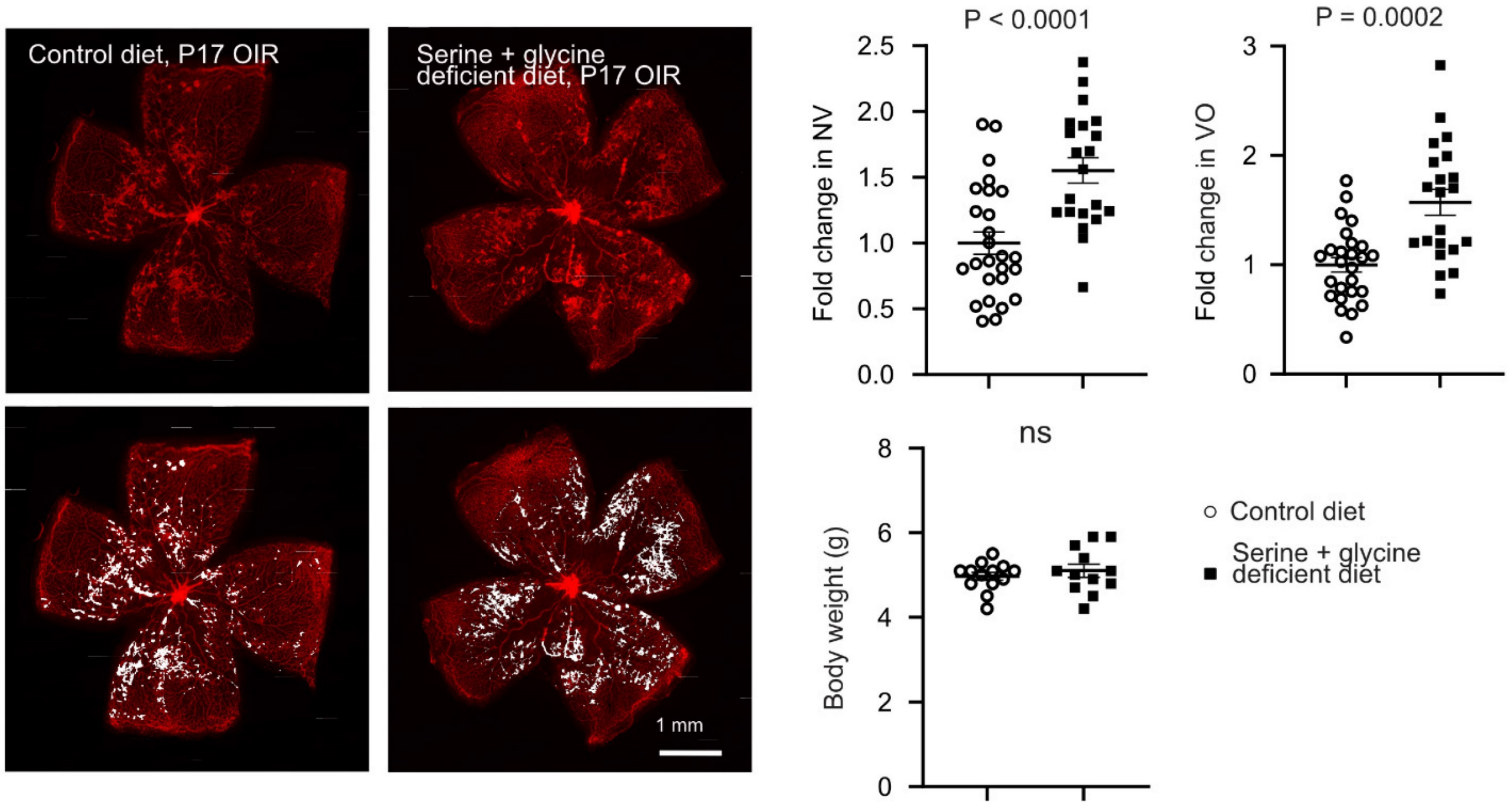
** Dietary shortage of serine and glycine worsened hypoxia-induced retinal neovascularization.** In mouse OIR, the nursing dam were fed Ser/Gly-deficient or control diet from P14 to P16 (during neovessel formation). At P17, At P17, retinal vaso-obliteration (VO, central area without red fluorescence) and neovascularization (NV, mid-peripheral area with high intensity of red fluorescence, highlighted in white) were examined. n = 21-26 retinas per group. Scale bar, 1 mm. Fold change was calculated relative to the average value of control diet. Unpaired t-test (or Welch's t-test) was used to compare the groups. ns, not significant.

To further examine the direct impact of serine on hypoxia-induced pathological retinal angiogenesis, we provided the nursing dam with a serine and glycine (interconvertible to serine) deficient diet from P14 when the retinal neovessel formation starts 16. Maternal dietary deficiency of serine and glycine worsened retinal neovascularization and delayed normal retinal re-vascularization (reflected by increased retinal vaso-obliterated area) at P17 (**Figure 2**). These findings further confirmed the protective role of serine supplementation against hypoxia-induced pathological retinal angiogenesis. The delayed retinal re-vascularization from serine/glycine deficiency was possibly due to the shortage of glycine. Retinal levels of serine were not significantly changed by serine supplementation or dietary serine/glycine deficiency (data not shown), possibly due to the fast depletion of serine, fasting status, and timing of tissue collection 36, 37.

### Serine supplementation via fatty acid oxidation suppressed hypoxia-induced neovascularization

**Figure 3:**
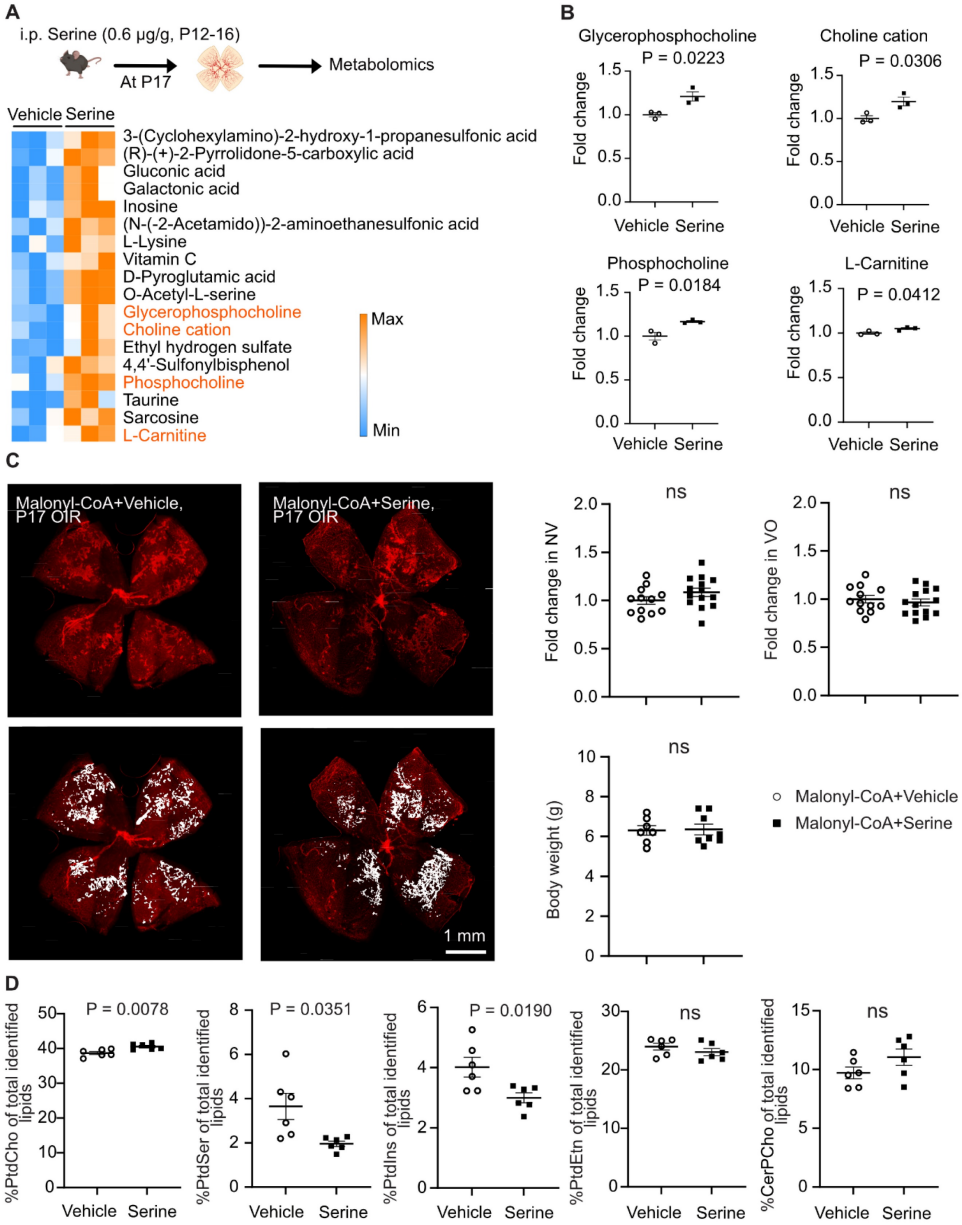
** Serine treatment via FAO protected hypoxic retinas.** (**A**) Metabolomic analysis revealed significantly altered retinal metabolites (*P* < 0.05) in P17 OIR neonates treated with serine (0.6 µg/g, i.p.) or vehicle from P12 to P16. Eight retinas from 4 mice were pooled to form one replicate (n = 1), with n = 3 replicates per group. A heatmap displaying the metabolites with significant changes (*P* < 0.05, unpaired t test) was generated using Morpheus. Lipid metabolites are highlighted in orange. The schematic was created using BioRender. (**B**) Metabolomic analysis identified an increase in lipid metabolites in serine-treated OIR retinas. Fold change was calculated and compared with vehicle group. Normality (histogram, QQ-plot, Sapiro-Wilk test) and variance (F-test) were confirmed. Unpaired t-test was then used to compare the groups. (**C**) Inhibition of fatty acid oxidation (FAO) attenuated the protective effect of serine in OIR mice. OIR neonates were treated with malonyl-CoA (15 mg/kg, i.p.) along with serine or vehicle from P12 to P16. At P17, retinal vaso-obliteration (VO, central area without red fluorescence) and neovascularization (NV, mid-peripheral area with high intensity of red fluorescence, highlighted in white) were examined. n = 12-14 retinas per group. Fold change was calculated relative to the average value of littermate vehicle controls. Unpaired t-test was used to compare the groups. ns, not significant. (**D**) Lipidomic analysis revealed serine treatment increased total retinal phosphatidylcholine (PtdCho) and decreased phosphatidylserine (PtdSer), phosphatidylinositol (PtdIns) levels in OIR retinas. No significant impact on phosphatidylethanolamine (PtdEtn) and sphingomyelin (CerPCho). Two retinas from one mouse were pooled to form one replicate (n = 1), with n = 6 replicates per group. The percentage of each lipid class relative to the total identified lipids was calculated. Normality (histogram, QQ-plot, Sapiro-Wilk test) and variance (F-test) were confirmed. Unpaired t-test or Welch's test was used to compare the groups. ns, not significant.

As hypoxic retinas exhibit metabolic dysfunction, which controls pathological retinal neovascularization 7, we next investigated if serine supplementation modulated the metabolism of OIR retinas. Metabolomic analysis showed significantly higher levels of L-carnitine (conjugating and transporting long-chain fatty acid (LCFA) into mitochondria for FAO), glycerophosphocholine, choline cation, and phosphocholine in serine- vs. vehicle-treated P17 OIR retinas (**Figure 3A-B**). To further validate the role of mitochondrial LCFA FAO in mediating the protective effect of serine against OIR, we co-administered serine or vehicle with inhibitors to block LCFA FAO. We used two commercially available inhibitors of mitochondrial LCFA transporter carnitine palmitoyltransferase 1A (CPT1A), including malonyl CoA, an endogenous inhibitor of CPT1A 38, and etomoxir. Notably, etomoxir may have off-target effects on mitochondrial complex I (the first protein complex of the mitochondrial respiration chain) and peroxisomal FAO 39-41. Serine suppression on neovascularization in OIR was not observed when cotreated with malonyl-CoA (**Figure 3C**) or etomoxir (**Figure S3**). These findings suggested that LCFA FAO was a key component in mediating serine protection against OIR.

As our metabolomic analysis showed serine increased retinal glycerophosphocholine, choline cation, and phosphocholine, we expected a potential influence of serine on retinal membrane lipids. Further lipidomic analysis of OIR retinas from mice treated with serine increased phosphatidylcholine (PtdCho) (**Figure 3D**), the major structural phospholipids in the retina; while the second most abundant structural phospholipid, phosphatidylethanolamine (PtdEtn), remains unchanged by serine administration. In contrast structural phospholipids, in OIR retina serine treatment decreased signaling phospholipids, phosphatidylserine (PtdSer) and phosphatidylinositol (PtdIns).

### Serine supplementation modulated mitochondrial metabolism in retinas with hypoxia-induced neovascularization

**Figure 4:**
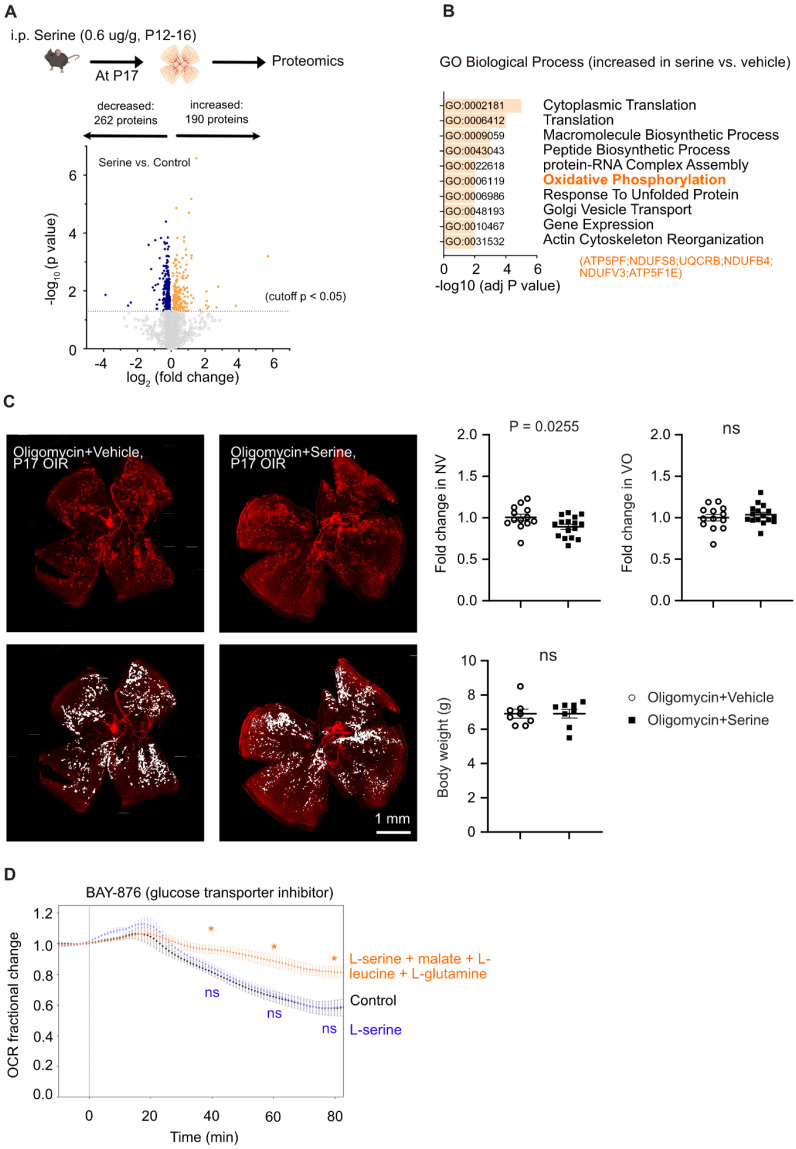
** Serine treatment preserved mitochondrial respiration in hypoxic retinas.** (**A**) Label-free LC-MS/MS-based proteomic analysis of P17 OIR retinas isolated from mice treated with serine (0.6 ug/g, i.p.) or vehicle control (vehicle; PBS, i.p.) from P12 to P16. Vehicle control group, n = 8; Serine, n = 8 (with two retinas pooled per mouse, n = 1). The volcano plot shows the differentially abundant proteins between serine- vs. control-treated OIR retinas. Each data point represents a unique protein, with the x-axis showing log_2_ (fold change) and the y-axis representing -log_10_ (p value). Out of 3,701 proteins identified (with at least two unique peptides), 452 proteins met the significant criteria (P < 0.05, adjusted p < 0.41). In serine- vs. control-treated group, 190 proteins were increased (highlighted in orange), and 262 proteins were decreased in abundance (highlighted in blue). (**B**) Gene ontology (biological process) enrichment analysis of proteins significantly enriched in serine- vs. vehicle- treated OIR retinas (190 proteins). The top 10 enriched pathways are shown, sorted by -log_10_ (adjusted P-value). The pathway for oxidative phosphorylation (OXPHOS) was highlighted in bold, and involved proteins were indicated in orange. (**C**) Retinal vaso-obliteration (VO, central area without red fluorescence) and neovascularization (NV, mid-peripheral area with high intensity of red fluorescence, highlighted in white) at P17 were examined in OIR pups treated with the mitochondrial ATP synthase inhibitor oligomycin (0.25 µg/g, i.p.), combined with either serine or vehicle from P12 to P16. n = 13-16 retinas per group. Fold change was calculated relative to the average value of littermate vehicle controls. Unpaired t-test was used to compare the groups. ns, not significant. (**D**) BaroFuse analysis of oxygen consumption rate (OCR), reflecting mitochondrial respiration, in P17 OIR retinas. The retinas were incubated in the presence of Krebs-Ringer Solution (1 mM glucose) (control medium), Krebs-Ringer Solution (1 mM glucose) supplemented with 10 mM serine or 10 mM serine + 5 mM malate + 1 mM L-leucine + 1 mM glutamine. The glucose transporter inhibitor BAY-876 (20 µM) was added to each medium to inhibit cellular glucose uptake. Oxygen (21%) was constantly supplied to the system. n = 3-4 retinas per group from two independent experiments. The factional changes in baseline OCR are shown. Unpaired t-test was used between each test and control medium. *P < 0.05; ns, not significant.

To further evaluate the mechanistic impact of serine supplementation on OIR retinas, we conducted a total proteome analysis. A total of 3701 proteins were characterized by at least two unique peptides, and 452 proteins (P < 0.05, adjusted p < 0.41) were significantly changed in abundance (**Figure 4A**). We found that serine vs. littermate vehicle control had an increased abundance of retinal proteins involved in OXPHOS (ATP5PF, NDUFS8, UQCRB, NDUFB4, NDUFV3, ATP5F1E) (**Figure 4B**), which is an efficient process for ATP generation in mitochondria. We have recently demonstrated a decrease in mitochondrial respiration in hypoxic OIR retinas, supported by a reduced oxygen consumption rate and a lower abundance of proteins involved in mitochondrial respiration 7. Moreover, supplementation with the mitochondrial fuel substrate pyruvate decreased retinal neovascularization in OIR 7. These findings suggested that improving mitochondrial energy production could serve as a novel and potential therapeutic approach for neovascular retinopathy. Our current proteomics and metabolomics data highlighted the role of serine in regulating mitochondrial activity. Specifically, serine supplementation increases 1) the abundance of proteins involved in OXPHOS and 2) the levels of L-carnitine, which supports mitochondrial FAO. To assess whether mitochondrial energy production plays a crucial role in serine's protective effect against OIR, we performed a cotreatment experiment using oligomycin, an inhibitor of ATP synthase, alongside serine or vehicle in OIR mice. Oligomycin specifically inhibits mitochondrial ATP synthase, a key enzyme in OXPHOS responsible for ATP production in mitochondria. Our results showed that pharmacological inhibition of ATP synthase significantly attenuated serine suppression of retinal neovascularization. There was about a 10% decrease in neovascularization in oligomycin plus serine- vs. oligomycin plus vehicle-treated group (**Figure 4C**), while there was about a 35% decrease in serine- vs. vehicle-treated group (**Figure 1A**). These findings suggested that mitochondrial metabolism plays an important role in mediating the protective effect of serine against OIR.

As serine had a significant impact on mitochondrial metabolism, we further tested if serine was a direct mitochondrial substrate of OIR retinas. We supplemented glucose-deprived P17 OIR retinas *ex vivo* with vehicle control, L-serine, or L-serine+malate+L-leucine+L-glutamine and measured OCR. Glucose deprivation was induced by blocking glucose transporters using BAY-876 7 and after a slight increase consistent with the Crabtree effect 42. OCR steadily decreased until about 60% of the baseline OCR remained. L-serine alone did not prevent the drop in OCR whereas L-serine + malate + L-leucine + L-glutamine significantly preserved OCR (**Figure 4D**), suggesting L-serine was not used as a direct mitochondrial substrate in OIR retinas *ex vivo*.

### Serine supplementation increased expression of genes involved in energy production in rod photoreceptors

Energy crisis in photoreceptors triggers retinal neovascularization, and photoreceptor-derived pro-angiogenic signals, in turn, drive the formation of retinal neovessels 43. To further explore the molecular responses of specific neural retinal cells to serine treatment, we conducted single-cell transcriptomics (10x sequencing) to examine retinal cell-specific gene profiles in P17 OIR retinas isolated from serine- (0.6 µg/g, i.p. P12 to P16) vs. vehicle control- (PBS) treated mice. Single-cell transcriptomics has been identified as a feasible approach to evaluate gene profiles in retinopathies 44 quantitatively. After quality controls, we uncovered a total of 32,493 cells from four control and four serine-treated mice.

**Figure 5:**
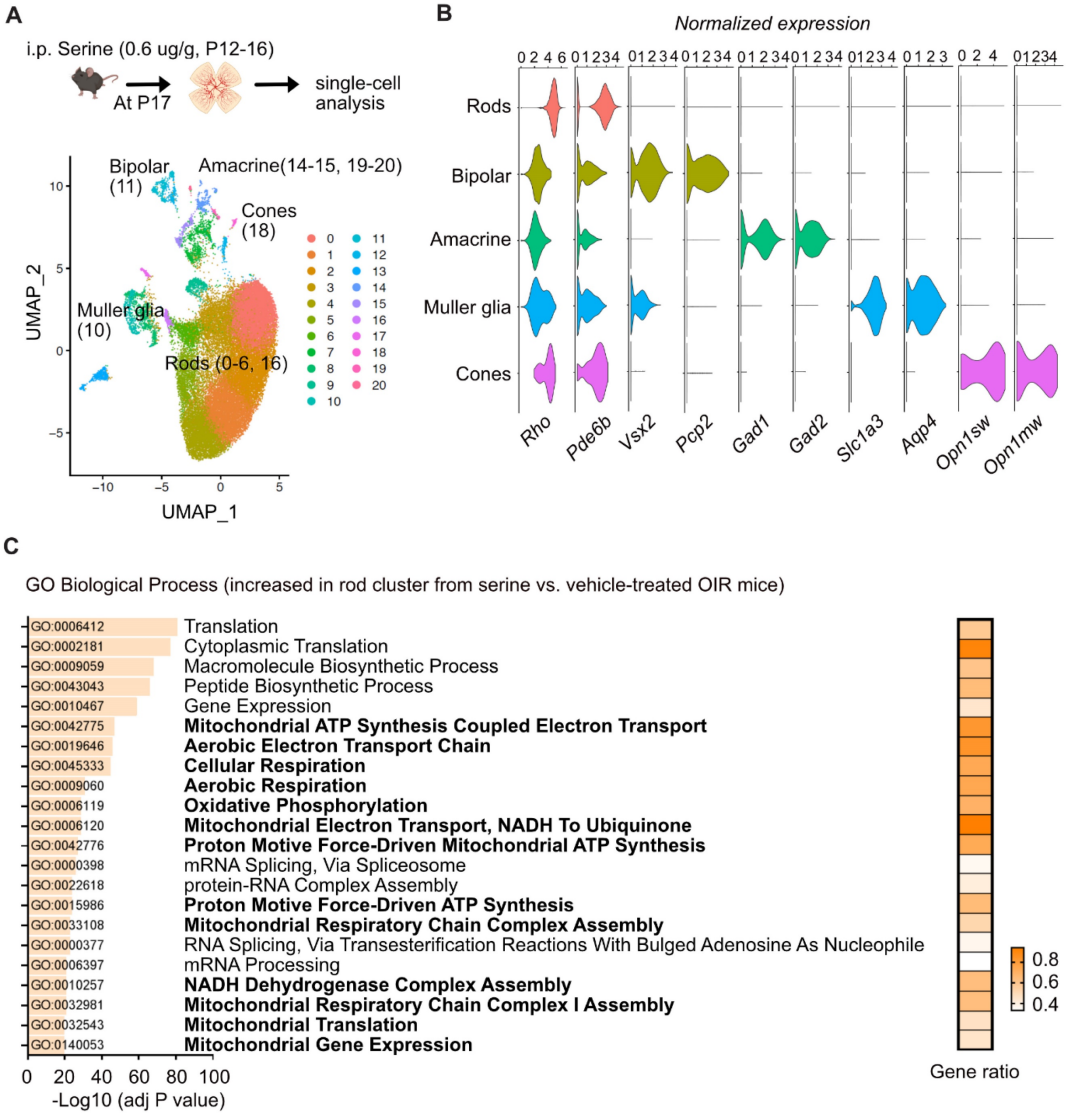
** Serine increased expression of genes involved in metabolism and decreased expression of genes involved in angiogenesis in rods.** (**A**) UMAP projection displaying the different color-coded retinal cell types in serine- vs. control- treated OIR pups. Vehicle control group, n = 4; Serine, n = 4 (with two retinas pooled per mouse, n = 1). (**B**) Dot plots showing the normalized expression of marker genes for different retinal cell types. Markers for rods, *Rho* and *Pde6b;* bipolar cells, *Vsx2* and *Pcp2;* Amacrine cells, *Gad1* and *Gad2*; Müller glia,* Slc3a1* and* Aqp4*; Cones, *Opn1sw* and *Opn1mw*. (**C**) Gene ontology (biological process) enrichment analysis of genes significantly differentially enriched in the rod cluster of serine- vs. control- treated OIR retinas. Pathways associated with mitochondrial respiration are highlighted in bold. The adjusted P-values for each term are shown in bar graphs (adjusted P < 0.001). The gene ratio for each pathway is represented in heatmap (right).

First, we have found 21 UMAP cell clusters with distinct expression profiles (**Figure 5A**). Cell types were identified by correlating expression profiles of individual cells with the profile from the Mouse Cell Atlas, independently validated by the expression of known markers, and collapsed into eight major cell types (**Figure 5B**). Cluster 7, 8, 9, 12, 13, and 17 had mixed expressions of cell marker genes and were excluded from analyses, while other clusters were combined into known cell types. We did not observe significant changes in the abundance of major cell types. However, tens to hundreds of differentially expressed genes (DEGs) were identified in the major cell types (rods, cones, Müller glia, bipolar cells, and amacrine cells) (**Table S3-7**).

Interestingly, we found that the expression of genes involved in mitochondrial respiration was significantly enriched with serine treatment in the rod cluster (**Figure 5C**). Rod photoreceptors are rich in mitochondria and highly energy-demanding 45, contributing to more than 60% of total retinal cells in mice 46. There was increased gene expression of *Ndufs8* (logFC = 0.0899, adjusted *P* < 0.001),* Uqcrb* (logFC = 0.1520, adjusted *P* < 0.001), *Ndufb4* (logFC = 0.1285, adjusted *P* < 0.001),* Ndufv3* (logFC = 0.1565, adjusted *P* < 0.001), *Atp5f1* (logFC = 0.1254, adjusted *P* < 0.001) in rod clusters from serine-treated mouse OIR retinas, in line with our proteome data (**Figure 4A, B**). We also observed increased expression of genes involved in mitochondrial respiration in bipolar cells and Müller glia. Meanwhile, serine treatment decreased the expression of genes involved in angiogenesis in the rod cluster, including *Hif1α* (logFC = -0.0111, adjusted *P* < 0.001), *Gsk3b* (logFC = -0.0162, adjusted *P* < 0.001), *Stat3* (logFC = -0.0532, adjusted *P* < 0.05), and *Jag1* (logFC = -0.1363, adjusted *P* < 0.01). Decreased total retinal levels of HIF-1α and phosphorylation of STAT3 (Tyr705) (reflects STAT3 activation) in serine- vs. vehicle-treated OIR mice was confirmed with western blot (**Figure S4**). No significant changes of these pro-angiogenic genes were observed in other retinal cell types (cones, Müller glia, bipolar cells, and amacrine cells). These findings suggested that serine tends to rescue rods from energy crisis and reduce the induction of pro-angiogenic signals.

**Figure 6:**
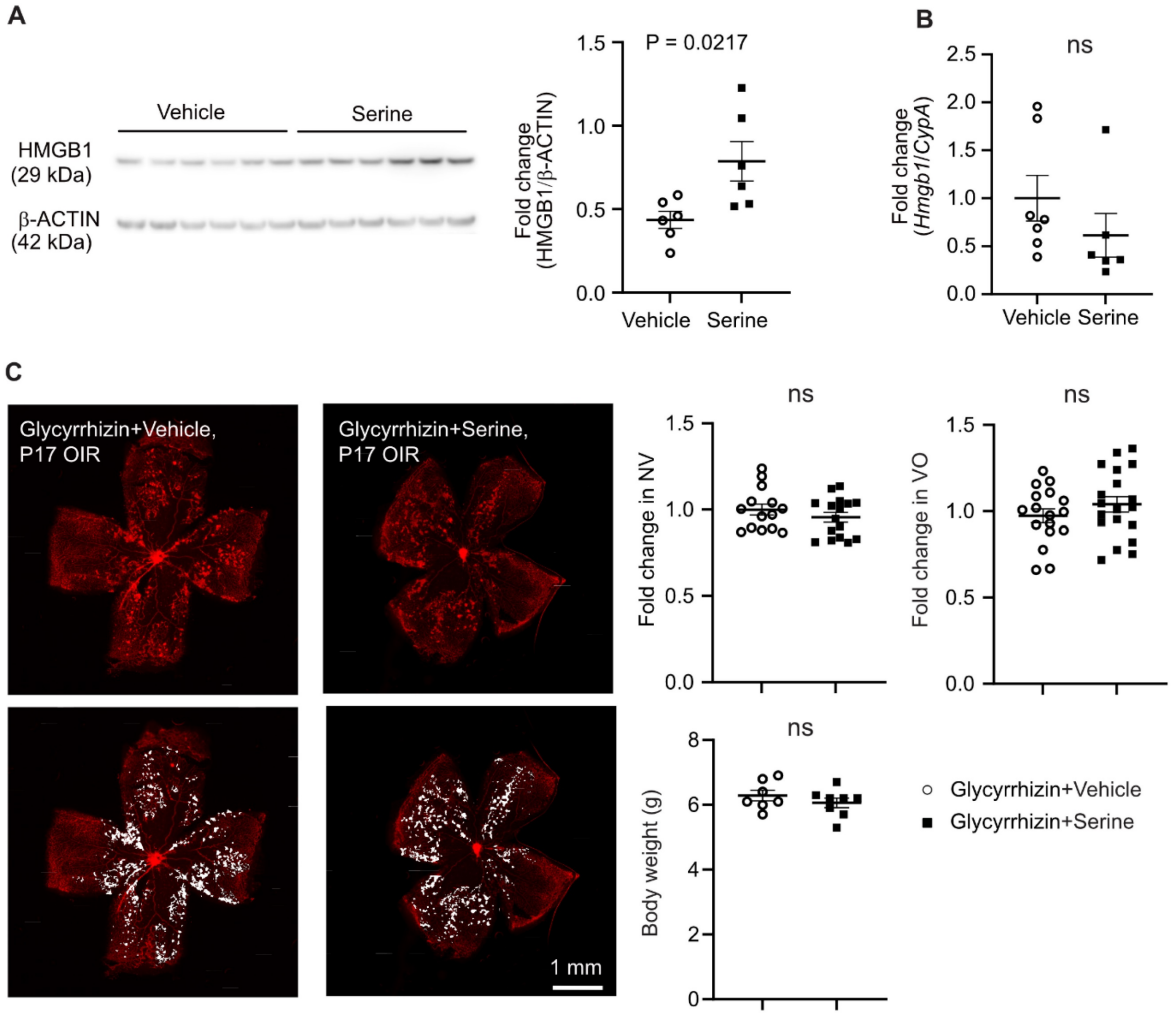
**HMGB1 mediated serine suppression of neovascularization in OIR.** (**A**) Protein levels of retinal HMGB1 from serine- vs. control- treated OIR mice were measured using western blot. β-ACTIN was used as an internal control. The band intensity of HMGB1 was normalized to β-ACTIN, and the fold change was calculated relative to the average value of the control group. Normality (QQ-plot) and F-test was first conducted. Unpaired t-test was used to compare the groups. Control, n = 6; Serine, n = 6 (with two retinas pooled per mouse, n = 1). (**B**) mRNA expression levels of *Hmgb1* were measured by qPCR. The relative value of *Hmgb1* over *CypA* was estimated and fold change was calculated referring to the average relative value of control group. Normality (QQ-plot) and F-test was first conducted. Mann-Whitney test was used to compare the groups. Control, n = 7; Serine, n = 6 (2 retinas from each mouse pooled as n = 1). ns, not significant. (**C**) All pups were treated with HMGB1 inhibitor glycyrrhizin (25 µg/g, i.p.), combined with either serine or vehicle, from P12 to P16. At P17, retinal vaso-obliteration (VO, central area without red fluorescence) and neovascularization (NV, mid-peripheral area with high intensity of red fluorescence, highlighted in white) were examined. n = 14-16 retinas per group. Fold changes were calculated relative to the average value of littermate vehicle controls. Normality (QQ-plot) and F-test was first conducted. Unpaired t-test or Mann-Whitney test was used to compare the groups. ns, not significant.

As serine supplementation increased levels of key mitochondrial metabolic proteins at transcriptional and translation levels, we further explored the potential transcriptional factor(s) modulating metabolism in serine-treated retinas with hypoxia-induced neovascularization. Increased proteins (proteomics) or genes in rod cluster (10x) were used for TF-Gene cooccurrence analysis in Enrichr. We found that high mobility group box 1 protein (HMGB1) was highly enriched in both proteomics and 10x datasets (adj P < 0.001). Additionally, proteomic analysis showed a significant increase in abundance of HMGB1 in retinas from serine-treated mice (logFC = 0.48, P < 0.01). HMGB1 deficiency causes mitochondrial fragmentation, decreased mitochondrial respiration, and ATP synthesis in embryonic fibroblasts 47. Hepatocytic loss of HMGB1 also downregulates the expression of key mitochondrial FAO genes, including *Cpt1a* 48. HMGB1 might be a potential mediator in serine modulation of retinal metabolism and suppression of neovascularization. In P17 OIR vs. normoxia control retinas, HMGB1 protein level was decreased, and mRNA level was unchanged (**Figure S5A-B**). Pharmaceutical inhibition of HMGB1 using glycyrrhizin (25 ug/g, i.p., a higher dose at 50 ug/g caused increased mortality in mouse neonates) did not affect OIR pathology (**Figure S5C**), possibly due to the decreased HMGB1 in OIR retinas, and therefore further suppression did not exacerbate the vascular pathology. We also confirmed the increase of retinal HMGB1 protein levels with serine treatment using western blot (**Figure 6A**). There was no significant change in the mRNA level of retinal *Hmgb1* in serine- vs. vehicle-treated OIR mice (**Figure 6B**). Cotreatment with glycyrrhizin (25 ug/g, i.p.) attenuated serine suppression of retinal neovascularization (**Figure 6C**) and abolished serine inhibition of HIF-1α and phosphorylation of STAT3 (**Figure S6**), confirming that HMGB1 mediated serine protection against OIR pathology.

## Discussion

**Figure 7:**
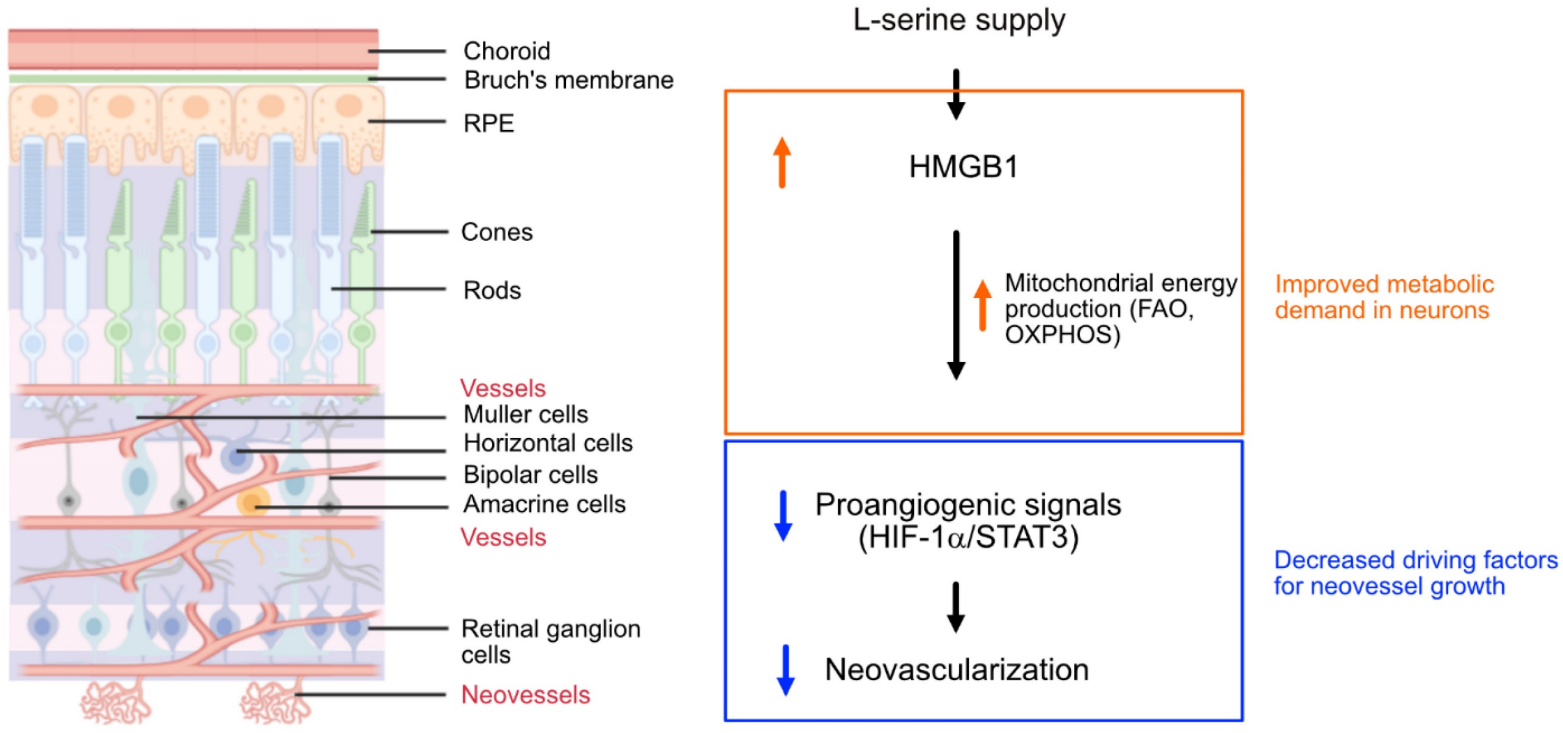
Schematics of the proposed pathway for serine-mediated suppression of retinal neovascularization. Serine upregulated HMGB1, which in turn enhanced mitochondrial metabolism (e.g. mitochondrial FAO, OXPHOS) to meet the metabolic demands of the developing neurons. This metabolic improvement led to a reduction in proangiogenic responses (HIF-1α induction and STAT3 activation) in the neural retina, thereby decreased the compensatory but pathological retinal neovascularization in hypoxic retina. The retinal structure diagram was generated using Biorender.com

Early cessation of retinal vessel growth or vessel loss in ROP and DR leads to hypoxia and nutrient deprivation in the local neural retina. This drives the formation of pathological retinal neovessel growth to address the unmet need of oxygen and nutrients 2, 49-51. Although we found that supplemented serine was not a direct mitochondrial energy source in isolated hypoxic retinas with neovascularization, our results suggested that mitochondrial FAO might be key in mediating serine protection against neovascularization. Serine supplementation also increased the abundance of proteins involved in OXPHOS in hypoxic retinas and predominantly improved the expression of metabolic genes in rod photoreceptors. We have recently reported that mitochondrial respiration decreased in hypoxic retinas with neovascularization and supplementing mitochondrial fuel substrate (pyruvate) improved retinal metabolism and decreased neovascularization 7. We therefore speculated that by meeting the metabolic demand of photoreceptors through serine supplementation in hypoxic retinas, the need for retinal vessel growth compensation was reduced. This, in turn, reduces pro-angiogenic factors hence dampened retinal neovascularization (**Figure 7**). However, we acknowledge that other potential mechanisms underlying serine suppression of retinal neovascularization as there was increased phospholipids, a key component of retinal membrane composition, in serine vs. vehicle (PBS)-treated OIR retinas.

Amino acids have been implicated in hypoxic retinal disorders, with altered serine and glycine metabolism suggested to correlate with ROP and DR 9-11. Serine emerges as a potential target, as patients with macular telangiectasia, with subretinal neovascularization, exhibit low circulating levels of serine and genetic defects of* PHGDH* 13, 14. Our current findings indicate that systemic serine supplementation decreased hypoxia-induced retinal neovascularization, highlighting the potential therapeutic approach of serine supplementation to treat ROP and diabetic retinopathy.

Serine maintains mitochondrial respiration in retinal Müller glia and retinal endothelial cells 52, 53, suggesting that serine could regulate retinal metabolism. We found that mitochondrial FAO was essential in promoting systemic serine to suppress hypoxia-induced retinal neovascularization. Further investigation into the role of mitochondrial FAO in controlling retinal neovascularization could better elucidate the mechanisms underlying disease progression. Moreover, serine serves as an important precursor of phospholipids, nucleotides, and the neurotransmitters glycine and D-serine in the brain 54. In our study, increased retinal levels of glycerophosphocholine, choline cation, and phosphocholine were detected in serine-treated hypoxia-exposed mouse retinas. Phosphocholine is a precursor of phosphatidylcholine, a major component of cell membranes. Lipidomic analysis confirmed that serine treatment in OIR retinas increased phosphatidylcholine, while phosphatidylserine and phosphatidylinositol decreased. Rod photoreceptor outer segments mainly consist of glycerophospholipids (> 90% by weight), and the phospholipids are dominated by phosphatidylcholine (∼30-40 mol%) 55. In developing mouse retinas, there is a continuous increase in the total levels of phosphatidylcholine and phosphatidylethanolamine 56. Notably, the exposure of phosphatidylserine on the cell surface is a marker of apoptosis 57. The phosphatidylinositol 3-kinase-Akt pathway plays a crucial role in regulating cell proliferation, apoptosis, and growth 58, and inhibition of Akt during hypoxia suppresses retinal neovascularization in OIR models 59. Our findings on phospholipid composition suggest that serine supplementation may help maintain retinal neuronal health during the hypoxic phase of OIR, potentially improving photoreceptor function and structure, which are often severely compromised in this condition 60.

In addition to directly improving the transport of mitochondrial fuel substrate by increasing L-carnitine, we also found that serine treatment increased the levels of proteins involved in OXPHOS. Through single-cell transcriptomics, we found that serine increased the expression of genes involved in mitochondrial respiration, predominantly in rod photoreceptors, which have a high density of mitochondria. While serine treatment appeared to improve energy production in these rod photoreceptors, we noted a reduced induction of genes associated with angiogenesis, including *Hif1α, Stat3, Jag1,* and* Gsk3β*. HIF1α upregulates the levels of VEGF, a significant contributor to hypoxia-induced retinal neovessel growth 61. STAT3-mediated activation of miR-21 promotes retinal neovascularization in mouse OIR 62. GSK3β positively regulates pro-inflammatory signals 63, which can trigger neovascularization in hypoxic retinas. Notably, inhibiting GSK3β has been shown to reduce retinal neovascularization and degeneration 64. Additionally, Jagged 1 protein (encoded by *Jag1*) mediates IL-33-induced retinal endothelial cell sprouting and neovascularization 65. Taken together, these findings suggest that serine treatment attenuated the pro-angiogenic signaling in rod photoreceptors, thereby decreasing the formation of retinal neovessels.

HMGB1 has been suggested as a potential transcriptional factor mediating the effects of serine in the suppression of retinal neovascularization. However, the role of HMGB1 in retinal health varies across different animal models. In a mouse model exposed to ischemia/reperfusion, inhibition of HMGB1 using glycyrrhizin helped mitigate the reduction in retinal thickness 66. In a rat model of glaucoma, treatment with an anti-HMGB1 antibody improved the survival of retinal ganglion cell 67. Conversely, mice with acute retinal detachment induced by sodium hyaluronate, loss of HMGB1 in rods accelerated retinal degeneration 68, 69. These findings suggest that the impact of HMGB1 on retina might be position-dependent. HMGB1 is primarily located in the nucleus but, under stress conditions, it translocates to the cytoplasm and is subsequently released into the extracellular space 70. In the nucleus, HMGB1 regulates chromosome structure and function, while in the cytoplasm, it supports autophagy. Intracellular HMGB1 plays a role in regulating fatty acid oxidation 48, which helps restore photoreceptor function through OXPHOS 71. Once released, HMGB1 acts as a mediator of proinflammatory responses 70. We here found that HMGB1 was deceased in OIR vs. normoxia retinas. Serine supplementation, via increasing HMGB1 protein levels, suppressed proangiogenic signaling pathways (HIF-1α/STAT3). Our ongoing research will further investigate the role of serine metabolism, the translational regulation of HMGB1, and the interactions between HMGB1 and mitochondrial activities in OIR retinas. Additionally, we aim to explore how mitochondrial FAO controls OIR pathology.

### Limitations

There are limitations when using mouse models to mimic human disease conditions. Specifically, the full-term mouse pups used in the OIR model cannot fully capture the characteristics of ROP in preterm infants. In preterm infants, low serine levels were found with low gestational age (and high risk for retinopathy of prematurity). In contrast, in the OIR mouse model, circulating and retinal levels of serine were induced, possibly as a compensatory response in full-term pups. Moreover, the OIR model does not fully replicate diabetic retinopathy due to differences in the underlying mechanisms, particularly between neonatal and adult mice. Nevertheless, despite these differences, the OIR model partially mimics the hypoxic and nutrient-deprived aspects of retinal neovascularization and provides a valuable system for investigating the mechanistic aspects of the stressed retinas directly.

*In vitro* analysis of isolated retina tissues faces their own set of challenges, as these tissues lack capillary flow and rely on diffusion to supply O_2_ to the cells. Although the use of a flow system (the BaroFuse), continuously supplies oxygenated media, there exists a gradient of the O_2_ from the surface to the core of the tissue. The steepness of this gradient is a complex function of the ratio of diffusion rate and tissue thickness, and the rate of OCR and its dependence on O_2_ concentration. Since 21% O_2_ (760 mmHg) was dissolved in the Krebs-Ringer solution, some of the cells are exposed to supraphysiologic concentrations of O_2_, and some of the cells at the core may experience lower than physiologic concentrations, the exact profile is technically difficult to measure. We cannot rule out the possibility that serine would serve as a fuel that supports OXPHOS at either lower or higher concentrations of O_2_, and we did not carry out effects of serine on OCR at different concentrations of O_2_.

Moreover, our current findings showed that oligomycin (0.25 µg/g) did not completely attenuate the serine suppression of retinal neovascularization. However, further increases in the dose of oligomycin (0.5 µg/g) affected postnatal weight gain and high death rate in neonatal pups. We therefore could not determine if serine protection against OIR was exclusively mediated by improving mitochondrial energy production.

Last but not least, our 10x analysis was dominated by rod cells. This high abundance of rods resulted in a relatively low number of bipolar cells, amacrine cells, Müller glia, and cones, thus limiting the statistical power to detect DEGs in these cell types. Indeed, most DEGs were detected in rods, consistent with the larger population of rods in the retina as compared to other cell types and thus higher statistical power. Furthermore, six out of twenty cell clusters were mixed with rods and were technically removed to ensure the purity of analyzed cell types. Given our primary focus on retinal metabolism, we did not deplete rods to enrich other cell types before library preparation. Despite this limitation, we were still able to detect tens to hundreds of DEGs in bipolar cells, amacrine cells, Müller glia, and cones. Future studies could consider depleting rods to enrich other cell types, which would likely improve statistical power for these specific cell populations.

## Conclusion

In summary, our data highlighted a protective role of serine supplementation in hypoxic neural retina, possibly by enhancing mitochondrial metabolism, thereby preventing pathological responses that lead to retinal neovascularization. Additionally, serine may improve photoreceptor health by promoting phospholipid synthesis during the hypoxic phase of OIR. These findings warrant further investigation, as targeting retinal metabolism through adequate nutrient supply could offer a promising therapeutic approach to prevent and treat neovascular disorders. A better understanding of how FAO regulates retinal neovascularization is also intriguing for uncovering disease mechanisms.

## Supplementary Material

Supplementary figures and tables 1-2.

Supplementary table 3.

Supplementary table 4.

Supplementary table 5.

Supplementary table 6.

Supplementary table 7.

## Abbreviations

CPT1A: Carnitine palmitoyltransferase 1A
DEGs: Differentially expressed genes
DR: Diabetic retinopathy
FAO: Fatty acid oxidation
HMGB1: High mobility group box 1
LCFA: Long-chain fatty acid
LC-MS: liquid chromatography-mass spectrometry
OCR: Oxygen-consumption rate
OIR: Oxygen-indcued retinopathy
OXPHOS: Oxidative phosphorylation
P: Postnatal
PHGDH: phosphoglycerate dehydrogenase
PtdCho: Phosphatidylcholine
PtdEtn: Phosphatidylethanolamine
Ptdlns: Phosphatidylinositol
PtdSer: Phosphatidylserine
ROP: Retinopathy of prematurity
VEGF: Vascular endothelial growth factor
