# Supplementary material for: Serine supplementation suppresses hypoxia-induced pathological retinal angiogenesis
Source: Theranostics. 2025 Apr 9;15(11):5087–105. doi: 10.7150/thno.105299 (PMC12036884; doi:10.7150/thno.105299)
Supplement: Supplementary file 1 — Supplementary figures and tables 1-2. [file thnov15p5087s1.pdf]

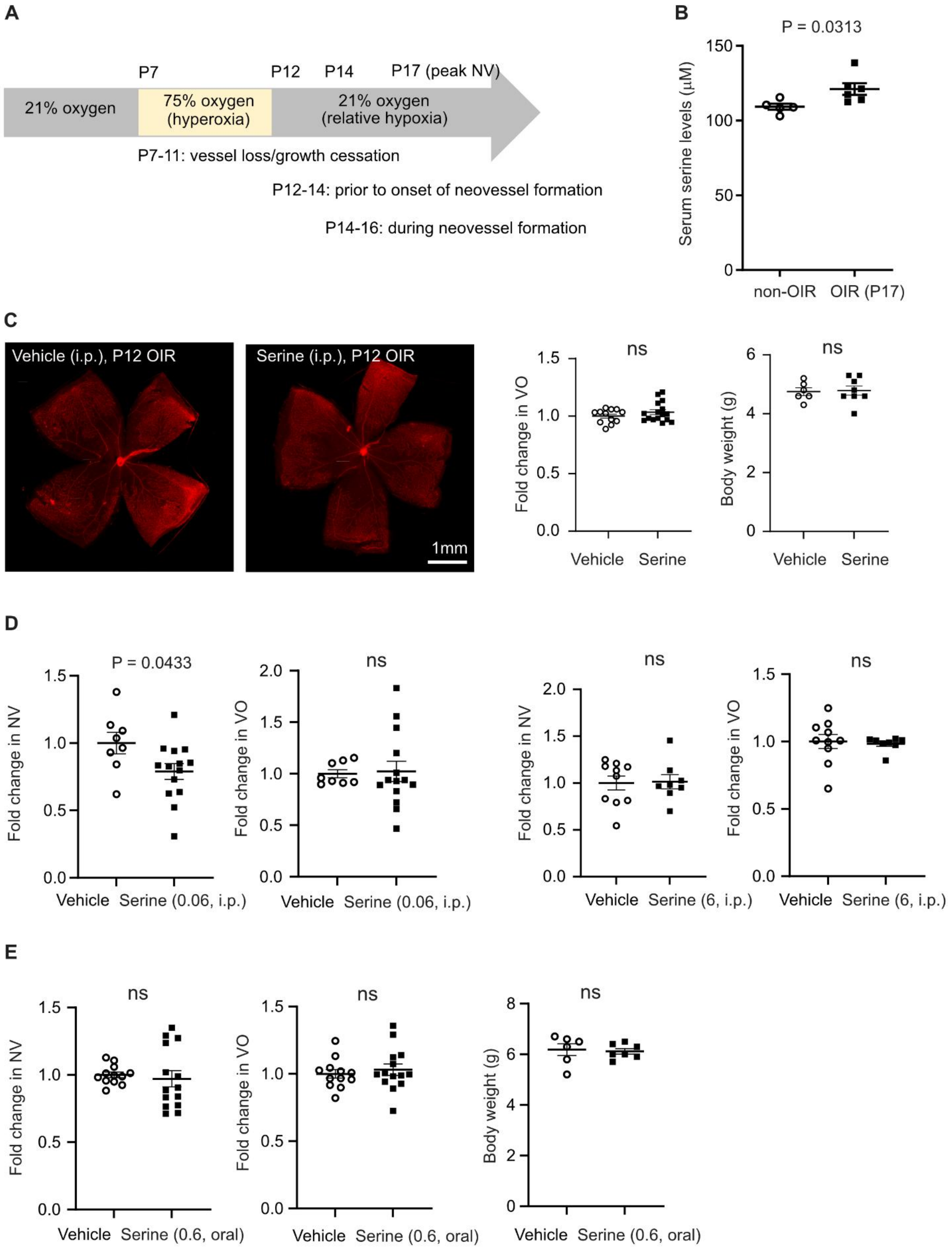
A
P7
P12
P14
P17 (peak NV)
21% oxygen
75% oxygen (hyperoxia)
21% oxygen (relative hypoxia)
P7-11: vessel loss/growth cessation
P12-14: prior to onset of neovessel formation
P14-16: during neovessel formation
B
P = 0.0313
Serum serine levels (μM)
non-OIR
OIR (P17)
C
Vehicle (i.p.), P12 OIR
Serine (i.p.), P12 OIR
1mm
ns
Fold change in VO
Body weight (g)
Vehicle
Serine
D
P = 0.0433
Fold change in NV
Vehicle
Serine (0.06, i.p.)
ns
Fold change in VO
Serine (6, i.p.)
E
Serine (0.6, oral)
Body weight (g)

Figure S1: Serine treatment did not affect hyperoxia-induced retinal vessel loss.
(**A**) Schematics of mouse OIR model. In mouse OIR, hyperoxia induced retinal vessel loss, followed by relative-hypoxia-induced retinal vessel proliferation (neovascularization, NV).
(**B**) Serum levels of L-serine in P17 OIR vs non-OIR mouse neonates were measured using DL-serine assay kit. n = 5-6 mice per group. Unpaired t-test.
(**C**) Serine (0.6 µg/g) or vehicle was administered by i.p. injection into OIR mice from P7 to P11. At P12, retinal vaso-obliteration (VO, central area without red fluorescence) was examined. n = 12-16 retinas per group. Scale bar, 1 mm. Ratio of change was calculated referring to the average value of littermate vehicle controls. Normality (histogram, QQ-plot, Sapiro-Wilk test) and variance (F-test) were confirmed. Unpaired t-test was used to compare the groups. ns, not significant.
(**D**) Serine (0.06 µg/g, or 6 ug/g) or vehicle was delivered by i.p. injection into OIR mice from P12 to P16. At P17, retinal neovascularization (NV, highlighted in white) and VO was examined. n = 8-14 retinas per group (0.06 µg/g, i.p.), n = 8-10 retinas per group (6 µg/g, i.p.), Scale bar, 1 mm. Fold change was calculated referring to the average value of littermate vehicle controls. No difference in body weight was found (0.06 ug/g: vehicle, 5.3 ± 0.1 g, serine 5.3 ± 0.1 g; 6 ug/g: vehicle, 6.5 ± 0.2 g, serine 6.7 ± 0.1 g). Normality (histogram, QQ-plot, Sapiro-Wilk test) and variance (F-test) were confirmed. Unpaired t-test, or Welch's test, or Mann-Whitney test were used to compare the groups. ns, not significant.
(**E**) Serine (0.6 µg/g) or vehicle was orally delivered to OIR mice from P12 to P16. At P17, retinal NV and VO were examined. n = 12-14 retinas per group. Scale bar, 1 mm. Fold change was calculated referring to the average value of littermate vehicle controls. Normality (histogram, QQ-plot, Sapiro-Wilk test) and variance (F-test) were confirmed. Unpaired t-test, or Welch's test was used to compare the groups. ns, not significant.

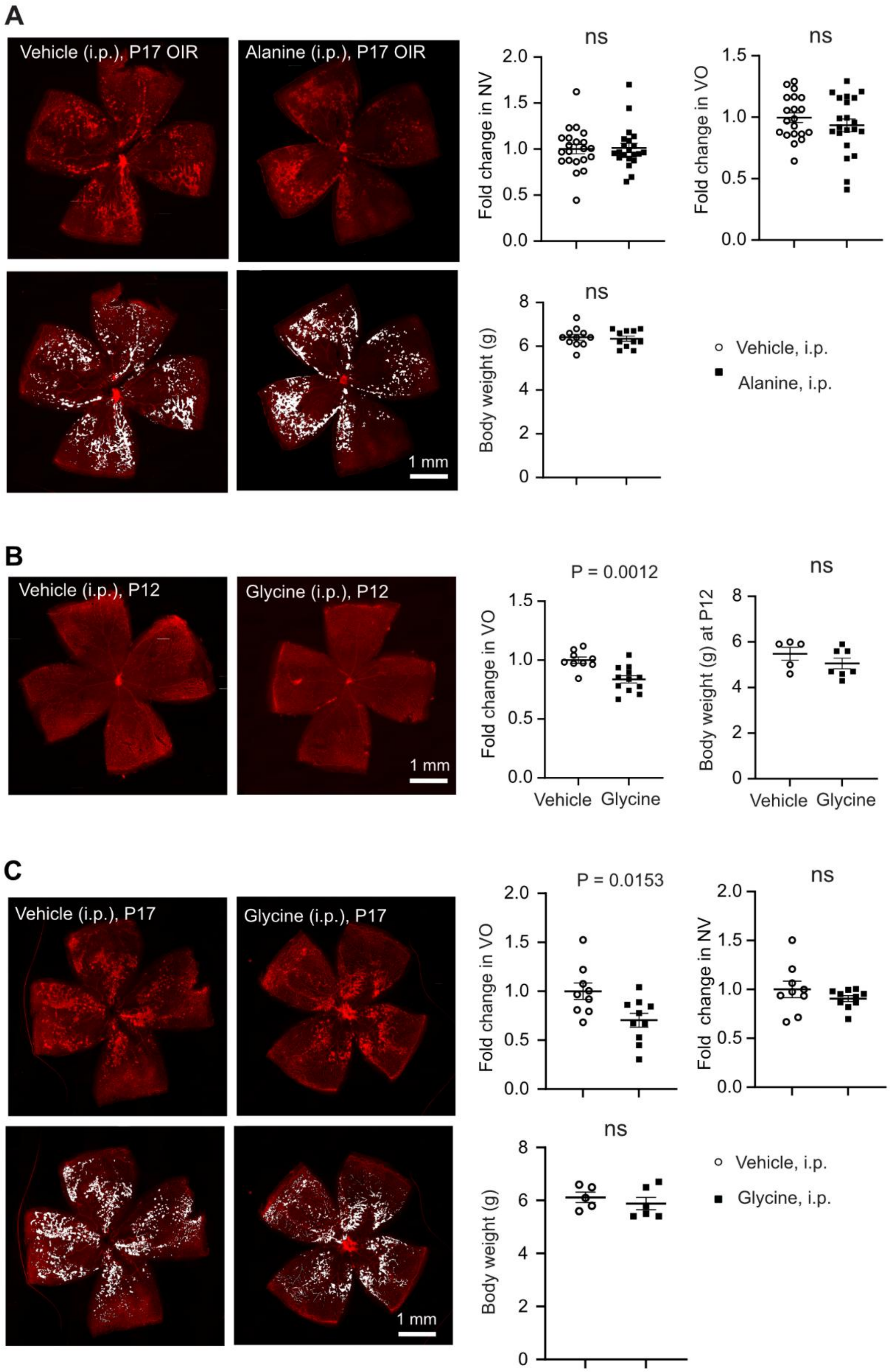
A
Vehicle (i.p.), P17 OIR
Alanine (i.p.), P17 OIR
1 mm
ns
Fold change in NV
2.0
1.5
1.0
0.5
0.0
ns
Fold change in VO
1.5
1.0
0.5
0.0
ns
Body weight (g)
8
6
4
2
0
Vehicle, i.p.
Alanine, i.p.
B
Vehicle (i.p.), P12
Glycine (i.p.), P12
1 mm
P = 0.0012
Fold change in VO
1.5
1.0
0.5
0.0
Vehicle
Glycine
ns
Body weight (g) at P12
8
6
4
2
0
Vehicle
Glycine
C
Vehicle (i.p.), P17
Glycine (i.p.), P17
1 mm
P = 0.0153
Fold change in VO
2.0
1.5
1.0
0.5
0.0
ns
Fold change in NV
2.0
1.5
1.0
0.5
0.0
ns
Body weight (g)
8
6
4
2
0
Vehicle, i.p.
Glycine, i.p.

Figure S2: Glycine treatment promoted retinal re-vascularization (reflected by decreased vaso-obliteration) in OIR mice.

(**A**) Alanine (2 µg/g) was delivered by i.p. injection into OIR mice from P12 to P16. Littermates were treated with vehicle. At P17, retinal vaso-obliteration (VO, central area without red fluorescence) and neovascularization (NV, mid-peripheral area with high intensity of red fluorescence, highlighted in white) were examined. n = 20-21 retinas per group. Scale bar, 1 mm. Fold change was calculated referring to the average value of littermate vehicle controls. Normality (histogram, QQ-plot, Sapiro-Wilk test) and variance (F-test) were confirmed. Unpaired t-test or nonparametric Mann-Whitney test was used to compare the groups. ns, not significant.

(**B-C**) Glycine (0.8 µg/g) was delivered by i.p. injection into OIR mice from P7 to P11 (**B**) or P12 to P16 (**C**). Littermates were treated with vehicle. At P12, P17, retinal vasculature was examined. n = 9-10 retinas per group. Scale bar, 1 mm. Unpaired t test. Fold change was calculated and compared with littermate vehicle controls. Normality (histogram, QQ-plot, Sapiro-Wilk test) and variance (F-test) were confirmed. Unpaired t-test or Welch's t-test was used to compare the groups. ns, not significant.

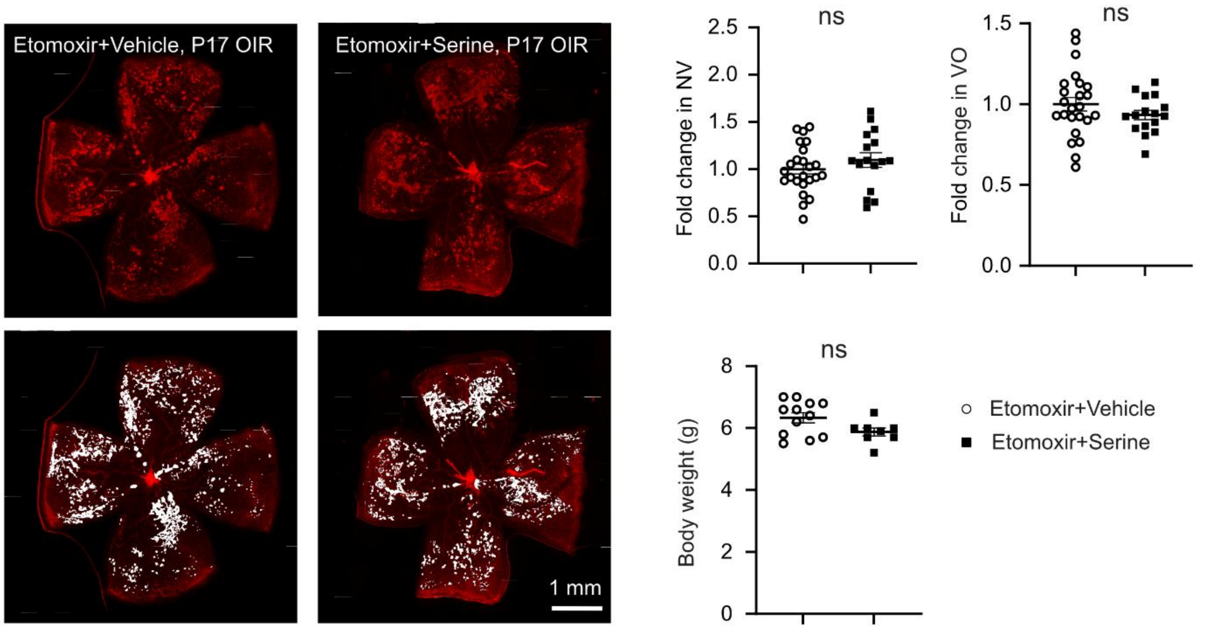

Figure S3: Blocking FAO using etomoxir attenuated serine protection against OIR. All pups were treated with etomoxir (4 mg/kg, i.p.) plus serine or vehicle from P12 to P16. At P17, retinal vaso-obliteration (VO, central area without red fluorescence) and neovascularization (NV, mid-peripheral area with high intensity of red fluorescence, highlighted in white) were examined.n = 16-24 retinas per group. Fold change was calculated relative to the average value of littermate vehicle controls. Normality (histogram, QQ-plot, Sapiro-Wilk test) and variance (F-test) were confirmed. Unpaired t-test or Welch's t-test was used to compare the groups. ns, not significant.

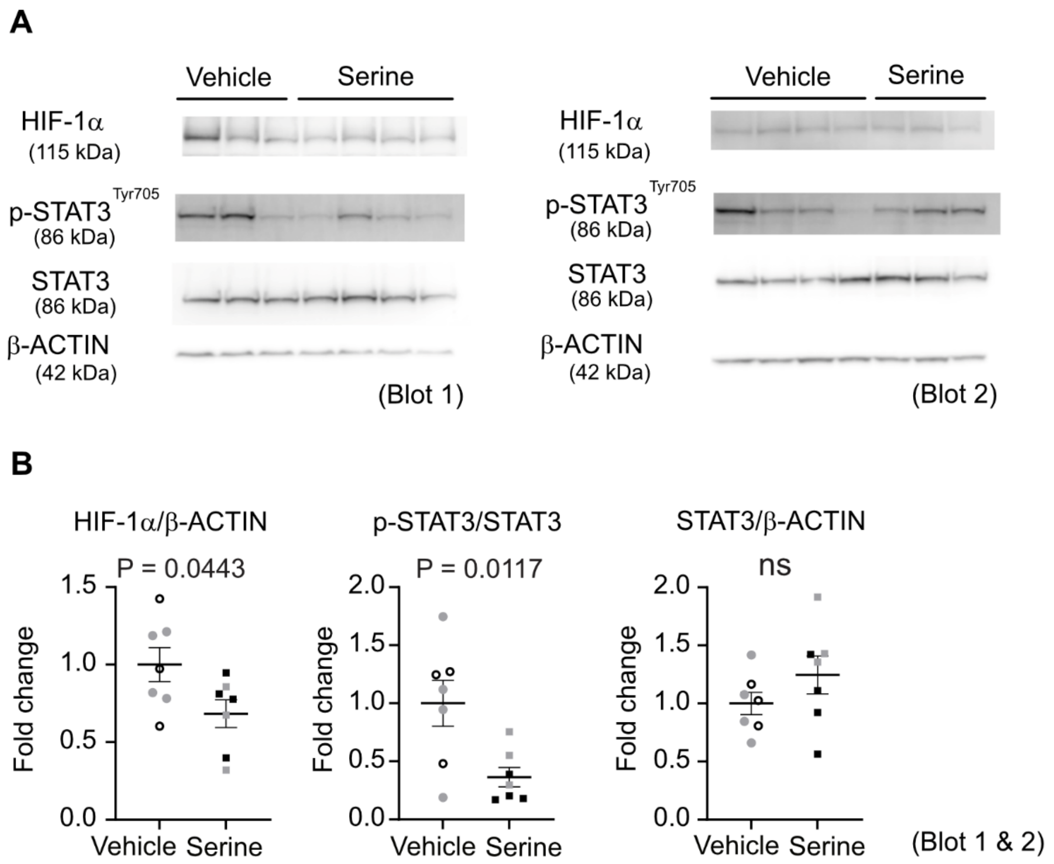

Figure S4: Serine treatment decreased pro-angiogenic signaling in OIR retinas.
(**A**) Protein levels of retinal HIF-1α, p-STAT3 (Tyr705), and STAT3 were measured in serine- vs. vehicle control-treated P17 OIR mice using western blot (two retinas were pooled per mouse as n = 1). β-ACTIN was used as an internal control. Western blot was conducted in two independent rounds using two independent mouse litters. For blot 1, n = 3 for vehicle and n = 4 for serine; for Blot 2, n = 4 for vehicle and n = 3 for serine (Vehicle, n = 7; Serine, n = 7; in total). (**B**) The fold changes in protein levels from Blot 1 (symbol in black) and Blot 2 (symbol in grey) were combined for analysis. The band intensities of target proteins were normalized to β-ACTIN levels, and the fold change was calculated relative to the average value of the vehicle controls for each blot. Normality (histogram, QQ-plot, Sapiro-Wilk test) and variance (F-test) were confirmed. Unpaired t-test was used. ns, not significant.

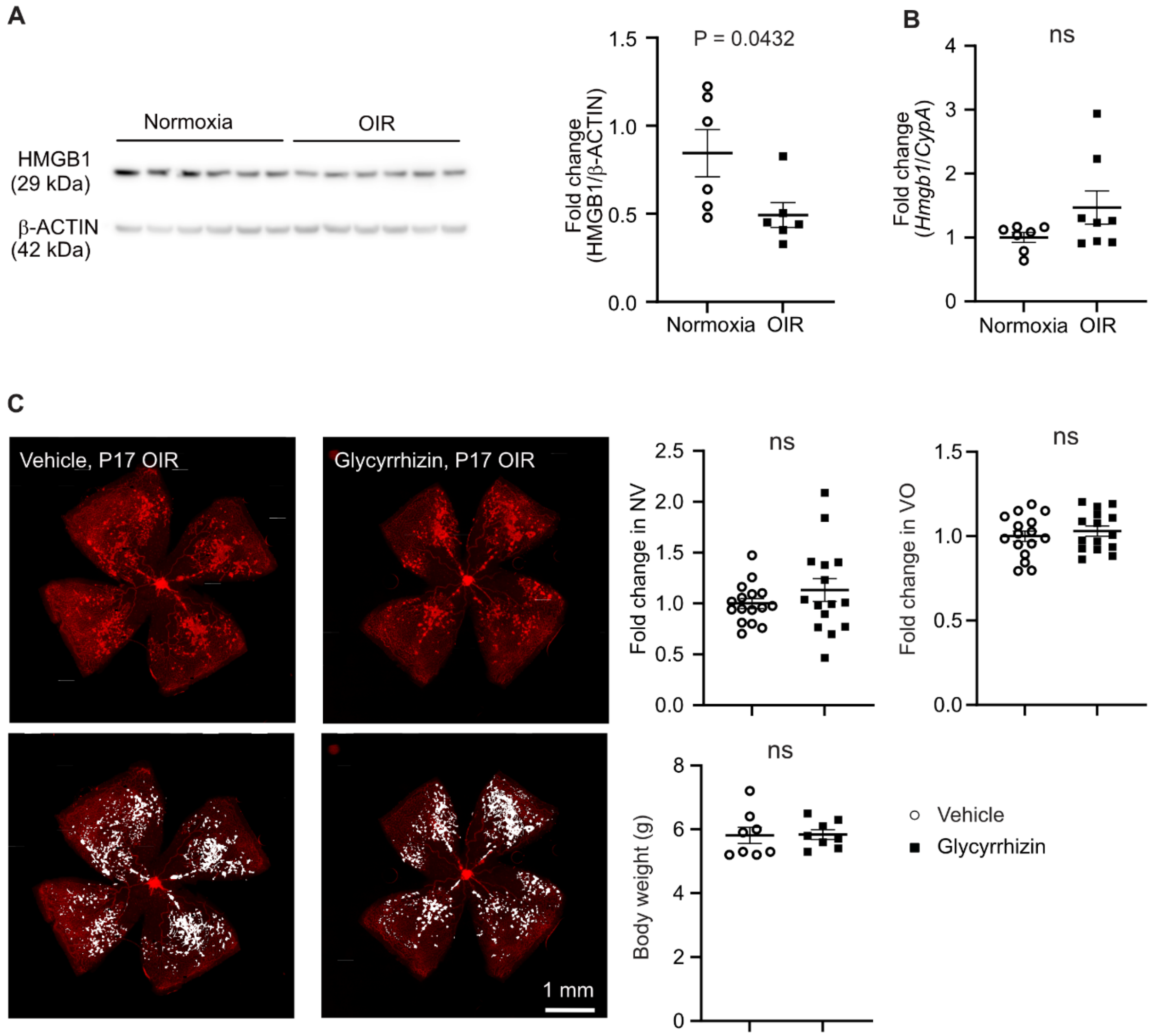

Figure S5: HMGB1 inhibitor glycyrrhizin did not affect neovascularization in OIR retinas.
(**A**) Protein levels of retinal HMGB1 were measured in P17 OIR vs. normal control (Normoxia) mice using western blot (two retinas from the same mouse were pooled as n = 1). β-ACTIN was used as an internal control. The band intensities of HMGB1 were normalized to β-ACTIN, and the fold change was calculated relative to the average value of the controls. Normality (histogram, QQ-plot, Sapiro-Wilk test) and variance (F-test) were confirmed. Unpaired t-test was used to compare the groups. Normoxia, n = 6; OIR, n = 6.
(**B**) mRNA expression levels of *Hmgb1* in P17 OIR vs. normal control (Normoxia) mice was examined with qPCR (two retinas from the same mouse were pooled as n = 1). The relative value of *Hmgb1* was divided by that of *CypA* for each sample. Fold change was calculated referring to the average relative value of control groups. Normality (histogram, QQ-plot, Sapiro-Wilk test) and variance (F-test) were confirmed. Mann-Whitney test was used to compare the groups. Normoxia, n = 7; OIR, n = 8. ns, not significant.
(**C**) OIR mouse pups were treated with HMGB1 inhibitor glycyrrhizin (25 µg/g, i.p.) or vehicle (DMSO) from P12 to P16. At P17, retinal vaso-obliteration (VO, central area without red fluorescence) and neovascularization (NV, mid-peripheral area with high intensity of red fluorescence, highlighted in white) were examined. n = 15-16 retinas per group. Fold change was calculated relative to the average value of littermate vehicle controls. Normality (histogram,

QQ-plot, Sapiro-Wilk test) and variance (F-test) were confirmed. Unpaired t-test or Welch's test was used to compare the groups. ns, not significant.

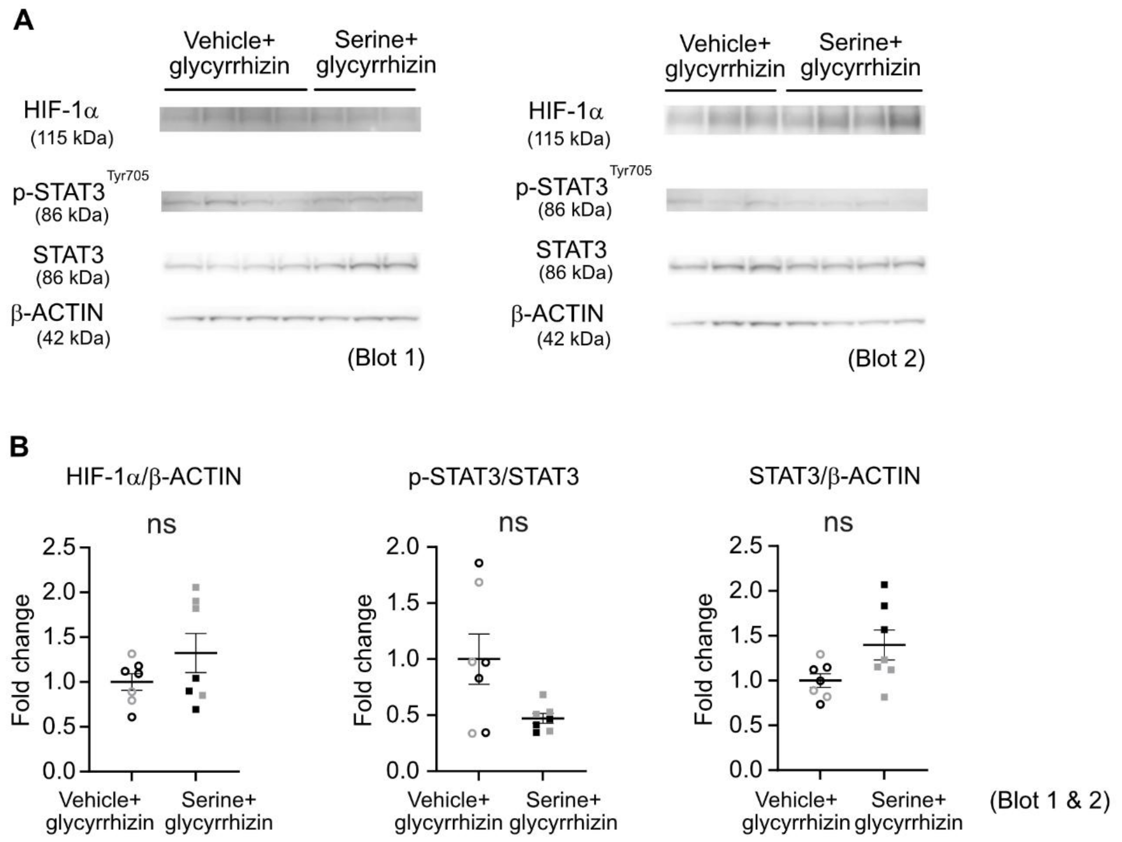

Figure S6: Blocking HMGB1 attenuated serine suppression of pro-angiogenic signaling in OIR retinas.

(**A**) Protein levels of retinal HIF-1α, p-STAT3 (Tyr705), and STAT3 were measured in serine+glycyrrhizin- vs. vehicle+glycyrrhizin- treated OIR mice using western blot (two retinas from the same mouse were pooled as n = 1). β-ACTIN was used as an internal control. Western blot was conducted in two independent rounds using two independent mouse litters. For blot 1, n = 4 for vehicle+glycyrrhizin and n = 3 for serine+glycyrrhizin; for Blot 2, n = 3 for vehicle+glycyrrhizin and n = 4 for serine+glycyrrhizin (Vehicle+glycyrrhizin, n = 7; Serine+glcyrrhizin, n = 7, in total).

(**B**) The fold changes in protein levels from Blot 1 (black symbols) and Blot 2 (grey symbols) were combined for analysis. The band intensities of target proteins were normalized to β-ACTIN, and the fold change was calculated relative to the average value of the vehicle controls on each blot. Normality (histogram, QQ-plot, Sapiro-Wilk test) and variance (F-test) were confirmed. Unpaired t-test was used. ns, not significant.

Teklad Custom Diet

TD.110839

Control AA Diet

Formula	g/Kg
L-Alanine	3.5
L-Arginine HCl	12.1
L-Asparagine	6.0
L-Aspartic Acid	3.5
L-Cystine	3.5
L-Glutamic Acid	40.0
Glycine	23.3
L-Histidine HCl, monohydrate	4.5
L-Isoleucine	8.0
L-Leucine	12.0
L-Lysine HCl	18.0
L-Methionine	8.2
L-Phenylalanine	7.5
L-Proline	3.5
L-Serine	3.5
L-Threonine	8.2
L-Tryptophan	1.8
L-Tyrosine	5.0
L-Valine	8.0
Sucrose	100.0
Corn Starch	381.18
Maltodextrin	150.0
Soybean Oil	80.0
Cellulose	50.0
Mineral Mix, AIN-93M-MX (94049)	35.0
Calcium Phosphate, monobasic, monohydrate	8.2
Vitamin Mix, AIN-93-VX (94047)	13.0
Choline Bitartrate	2.5
TBHQ, antioxidant	0.02

Footnote

Amino acid defined diet designed as a control for diets with adjusted levels of amino acids. Carbohydrate provided primarily by starch.

Key Features

+ **Amino Acid Defined Diet**
+ **Rodent**
+ **Control**

Selected Nutrient Information[1]

	% by weight	% kcal from
**Protein**	15.4	16.4
**CHO**	60.6	64.5
**Fat**	8.0	19.2
**Kcal/g**	**3.8**	

[1] Calculated values

[2] Protein based on N x 6.25

Teklad Diets are designed & manufactured for research purposes only.

Key Planning Information

+ *Products are made fresh to order*
+ Store product at 4°C or lower
+ Use within 6 months (applicable to most diets)
+ Box labeled with product name, manufacturing date, and lot number
+ Replace diet at minimum once per week
  More frequent replacement may be advised
+ Lead time:
  · 2 weeks non-irradiated
  · 4 weeks irradiated

Product Specific Information

+ 1/2" Pellet or Powder (free flowing)
+ Minimum order 3 Kg
+ Irradiation not advised
  · Contact a nutritionist for recommendations

Options (Fees Will Apply)

+ Rush order (pending availability)
+ Irradiation (see Product Specific Information)
+ Vacuum packaging (1 and 2 Kg)

Speak With A Nutritionist

+ **(800) 483-5523**
+ **askanutritionist@envigo.com**

Contact Us

Obtain Pricing · Check Order Status

+ **teklad@envigo.com**
+ **(800) 483-5523**

International Inquiry *(Outside USA or Canada)*

+ **askanutritionist@envigo.com**

Place Your Order (USA & Canada)

Please Choose One

+ **www.envigo.com/teklad-orders**
+ **tekladorders@envigo.com**
+ **(800) 483-5523**
+ **(608) 277-2066** *facsimile*

© 2016 Envigo

07/01/16

Supplemental Table 1: Nutrient composition of serine/glycine control diet.

Teklad Custom Diet

TD.150056

Gly and Ser Deficient AA Diet, R

ENVIGO

Formula	g/Kg
L-Alanine	**6.123**
L-Arginine HCl	**12.1**
L-Asparagine	**10.496**
L-Aspartic Acid	**6.123**
L-Cystine	**3.5**
L-Glutamic Acid	**69.976**
L-Histidine HCl, monohydrate	**4.5**
L-Isoleucine	**8.0**
L-Leucine	**12.0**
L-Lysine HCl	**18.0**
L-Methionine	**8.2**
L-Phenylalanine	**7.5**
L-Proline	**6.123**
L-Threonine	**8.2**
L-Tryptophan	**1.8**
L-Tyrosine	**5.0**
L-Valine	**8.0**
Sucrose	**100.0**
Corn Starch	**365.539**
Maltodextrin	**150.0**
Soybean Oil	**80.0**
Cellulose	**50.0**
Mineral Mix, AIN-93M-MX (94049)	**35.0**
Calcium Phosphate, monobasic, monohydrate	**8.2**
Vitamin Mix, AIN-93-VX (94047)	**13.0**
Choline Bitartrate	**2.5**
TBHQ, antioxidant	**0.02**
Red Food Color	**0.1**

Footnote

Isonitrogenous modification of TD.110839 to remove glycine and serine. Color coded red.

Key Features

+ **Amino Acid Defined Diet**
+ **Isonitrogenous**
+ **Deficient Gly & Ser**
+ **Color Coded Red**

Selected Nutrient Information[1]

	% by weight	% kcal from
**Protein**	15.4	16.6
**CHO**	59.1	63.9
**Fat**	8.0	19.5
**Kcal/g**	**3.7**	

[1] Calculated values

[2] Protein based on N x 6.25

Teklad Diets are designed & manufactured for research purposes only.

Key Planning Information

+ *Products are made fresh to order*
+ Store product at 4°C or lower
+ Use within 6 months (applicable to most diets)
+ Box labeled with product name, manufacturing date, and lot number
+ Replace diet at minimum once per week
  More frequent replacement may be advised
+ Lead time:
  · 2 weeks non-irradiated
  · 4 weeks irradiated

Product Specific Information

+ 1/2" Pellet or Powder (free flowing)
+ Minimum order 3 Kg
+ Irradiation not advised
  · Contact a nutritionist for recommendations

Options (Fees Will Apply)

+ Rush order (pending availability)
+ Irradiation (see Product Specific Information)
+ Vacuum packaging (1 and 2 Kg)

Speak With A Nutritionist

+ **(800) 483-5523**
+ **askanutritionist@envigo.com**

Contact Us

Obtain Pricing · Check Order Status

+ **teklad@envigo.com**
+ **(800) 483-5523**

International Inquiry *(Outside USA or Canada)*

+ **askanutritionist@envigo.com**

Place Your Order (USA & Canada)

Please Choose One

+ **www.envigo.com/teklad-orders**
+ **tekladorders@envigo.com**
+ **(800) 483-5523**
+ **(608) 277-2066** *facsimile*

© 2015 Envigo

06/29/16

JBT

Supplemental Table 2: Nutrient composition of serine/glycine deficient diet.
